# Selective Electrosynthesis of Methanol from CO_2_ Over Cu/Cu_2_P_2_O_7_ Via the Formate Pathway

**DOI:** 10.1002/adma.202501021

**Published:** 2025-05-20

**Authors:** Hyunwoo Kim, Jihoe Lee, Sangseob Lee, Suhwan Park, Yongseok Lee, Giyeok Lee, Hyo Sang Jeon, Man Ho Han, Sunghwan Jin, Hyun‐Wook Lee, Aloysius Soon, Jongsoon Kim, Jungki Ryu

**Affiliations:** ^1^ School of Energy and Chemical Engineering Ulsan National Institute of Science and Technology (UNIST) Ulsan 44919 Republic of Korea; ^2^ Department of Energy Science Sungkyunkwan University (SKKU) Suwon 16419 Republic of Korea; ^3^ Department of Materials Science and Engineering Yonsei University Seoul 03722 Republic of Korea; ^4^ Sustainable Energy Research Division Korea Institute of Science and Technology (KIST) Seoul 02792 Republic of Korea; ^5^ Clean Energy Research Center Korea Institute of Science and Technology (KIST) Seoul 02792 Republic of Korea; ^6^ Department of Materials Science and Engineering Kangwon National University Samcheok 25913 Republic of Korea; ^7^ SKKU Institute of Energy Science and Technology (SIEST) Sungkyunkwan University (SKKU) Suwon 16419 Republic of Korea; ^8^ Graduate School of Carbon Neutrality Ulsan National Institute of Science and Technology (UNIST) Ulsan 44919 Republic of Korea; ^9^ Center for Renewable Carbon Ulsan National Institute of Science and Technology (UNIST) Ulsan 44919 Republic of Korea

**Keywords:** Cu/Cu_2_P_2_O_7_ catalyst, electrochemical CO_2_ reduction reaction, HCOOH pathway, methanol production

## Abstract

The electrochemical CO_2_ reduction reaction (CO2RR) to methanol offers an eco‐friendly approach to reducing carbon emissions while producing versatile liquid fuels and feedstocks. However, achieving high selectivity for methanol, especially at high current densities, remains challenging due to competing reactions that favor methane and hydrogen formation. Here, the tailored synthesis of Cu/Cu_2_P_2_O_7_‐based hybrid catalysts is reported for efficient and selective methanol production through the discharge of lithium‐ion batteries. The catalyst exhibits a Faradaic efficiency exceeding 50% in both H‐cells and gas‐diffusion electrode cells, achieving one of the highest reported methanol partial current densities of over 100 mA cm^−2^. Experimental and computational analyses reveal a synergistic effect between Cu nanoparticles with a predominant (111) surface and Cu_2_P_2_O_7_ nanoparticles, which enhances selective methanol production via the HCOOH intermediate pathway. These findings provide insights into designing cost‐effective electrocatalysts for selective methanol production.

## Graphical abstract

A Cu/Cu_2_P_2_O_7_‐based hybrid catalyst delivers over 50% Faradaic efficiency and >100 mA cm^−2^ partial current density for methanol electrosynthesis from CO_2_. The synergy between Cu (111) facets and Cu_2_P_2_O_7_ promotes HCOOH‐mediated pathways, offering a scalable route for sustainable liquid fuel production.

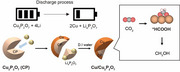

## 1 Introduction

Electrochemical CO_2_ reduction reactions (CO2RR) offer a promising route for mitigating carbon emissions^[^ ^1^ ^]^ while sustainably producing valuable chemicals,^[^ ^2^ ^]^ including methane,^[^ ^3^ ^]^ ethylene,^[^ ^4^ ^]^ methanol,^[^ ^5^ ^]^ and ethanol.^[^ ^6^ ^]^ Among these products, methanol has garnered particular attention due to its dual role as both a fuel and feedstock.^[^ ^7^ ^]^ Despite significant recent advances in designing efficient electrocatalysts with high selectivity for various hydrocarbons^[^ ^3^, ^4^ ^]^ and oxygenates,^[^ ^5^, ^6^ ^]^ developing catalysts with high selectivity, especially for methanol, remains challenging. This difficulty arises mainly due to competing reactions, such as CO2RR to methane^[^ ^8^ ^]^ and hydrogen evolution reactions (HER).^[^ ^9^ ^]^ Methanol and methane productions via CO2RR share key intermediates, such as ^*^HCO and ^*^HCHO,^[^ ^10^ ^]^ which can either be converted to methanol through successive hydrogenation^[^ ^11^ ^]^ or to methane through deoxygenation followed by hydrogenation.^[^ ^12^ ^]^ To address this, researchers have suggested that an optimal *H coverage on catalysts is crucial for selectively producing methanol.^[^ ^13^ ^]^ However, excessive ^*^H coverage can promote HER, lowering the Faradaic efficiency for methanol production. Recently, promising electrocatalysts for methanol production, such as Ag,S‐Cu_2_O/Cu,^[^ ^14^ ^]^ Rh_1_Cu_4_,^[^ ^15^ ^]^ and Cu/Au/NCF,^[^ ^16^ ^]^ have been reported. However, these often rely on expensive noble metals like Rh and Au, which are particularly effective for hydrogenation catalysis.^[^ ^17^ ^]^ Moreover, most of these catalysts show high methanol selectivity in non‐aqueous electrolytes, where limited proton availability and low ionic conductivity present substantial obstacles to practical applications.^[^ ^18^ ^]^

In this context, phosphate‐based materials emerge as promising catalyst candidates for selective methanol production. Phosphates offer advantageous properties for CO2RR, including polyprotic nature,^[^ ^19^ ^]^ exceptional chemical stability,^[^ ^20^ ^]^ and strong interaction with alkali metal ions like potassium ions,^[^ ^21^ ^]^ which can reduce the activation energy barrier of CO2RR while suppressing HER. Previous studies have reported phosphate‐based catalysts for selective formic acid production.^[^ ^22^ ^]^ However, despite the higher demand and value of methanol compared to formic acid, cost‐effective Cu and phosphate‐based electrocatalysts for methanol production have yet to be reported (Tables S1 and S2, Supporting information), although many Cu‐based thermocatalysts^[^ ^7b,c^ ^]^ for methanol production have been reported recently. This gap likely arises from the increased complexity of methanol production, which involves a six‐electron, six‐proton transfer process and requires optimal ^*^H coverage to promote successive hydrogenation while suppressing HER. Therefore, precise control over the structure, composition, and ^*^H coverage on phosphate‐based catalysts may be essential for designing selective methanol catalysts.

In this study, we report the tailored electrochemical synthesis of Cu‐ and phosphate‐based hybrid catalysts for selective methanol production via CO2RR. Cu pyrophosphate (Cu_2_P_2_O_7_) can be transformed into a mixture of Cu and Cu_2_P_2_O_7_ nanoparticles with precisely tuned compositions through the discharge process of Li‐ion batteries. Hybrid catalysts with the optimal composition exhibit high methanol selectivity and stability, achieving Faradaic efficiencies of 48.8% in 0.1 m KHCO_3_ and 70.1% in 0.1 m CsHCO_3_ in H‐cells, while maintaining stable operation for 48 h. The hybrid catalyst also sustained its activity even on a gas‐diffusion electrode (GDE), achieving the highest reported methanol partial current densities for Cu‐based catalysts without noble metals, exceeding 100 mA cm^−2^. In situ X‐ray absorption spectroscopy and density functional theory (DFT) calculations suggest that the coexistence of Cu nanoparticles with (111) surface and Cu_2_P_2_O_7_ nanoparticles enables selective methanol production through the HCOOH intermediate pathway. This study provides valuable insights into the design and optimization of cost‐effective electrocatalysts for efficient CO2RR, especially for selective methanol production.

## 2 Results and Discussion

### 2.1 Preparation and Characterization of the Hybrid Catalysts

**Figure 1:**
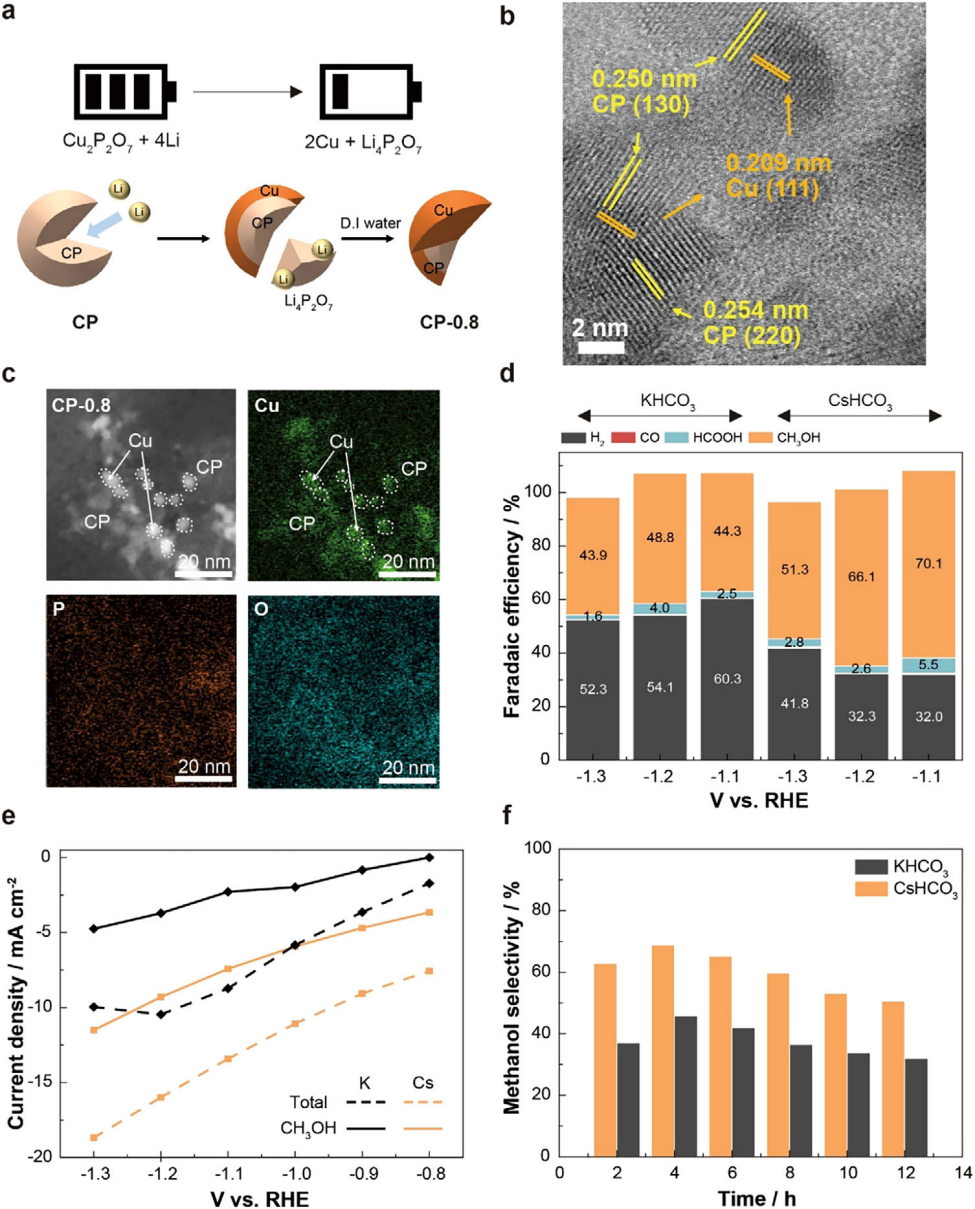
Synthesis and electrochemical CO2RR performance of Cu/CP hybrid catalysts. a) Schematic illustration of the preparation of Cu/CP hybrid catalysts via the battery discharge process, followed by washing with water. b,c) Transmission electron microscopy (TEM) (b) and elemental mapping images (c) of CP‐0.8. d,e) CO2RR performance of the hybrid catalyst: (d) Faradaic efficiency and (e) methanol partial current density of CP‐0.8 in 0.1 m KHCO_3_ or 0.1 m CsHCO_3_ electrolytes. f) Faradaic efficiency profile of CP‐0.8 for methanol production at −1.2 V versus RHE.

We synthesized nanoparticulate hybrid electrocatalysts composed of metallic Cu and Cu_2_P_2_O_7_ (CP) for selective methanol production, using pristine CP as a precursor. Unlike noble metals^[^ ^14^, ^15^, ^16^ ^]^ and Co,^[^ ^23^ ^]^ which are conventionally used in methanol production catalysts, Cu is both abundant and one of the most active elements for CO2RR. Additionally, phosphate offers beneficial properties for CO2RR, such as its polyprotic nature and strong interaction with alkali metal ions. Based on these properties, we hypothesized that nanocomposites of Cu and phosphates could be designed as effective CO2RR catalysts. CP, a promising next‐generation cathode candidate in Li‐ion batteries, was chosen as the catalyst precursor due to its ability to transform into a mixture of metallic Cu and Li_4_P_2_O_7_ during the discharge process^[^ ^24^ ^]^ (Cu_2_P_2_O_7_ + 4Li^+^ + 4e^−^ → 2Cu^0^ + Li_4_P_2_O_7_) (**Figure** **1**a; Figure S1, Supporting Information). To enhance electrical conductivity, carbon nanotubes (CNTs) were incorporated into the catalyst design. As Li_4_P_2_O_7_ is soluble in water, Cu/CP‐based hybrid materials with tailored compositions were synthesized by discharging CP at a specified voltage, followed by washing with water (Figures S2 and S3, Supporting Information). The hybrid catalyst discharged at X.X V is denoted as CP‐X.X. Electron microscopy confirmed the successful formation of Cu/CP nanocomposites (Figure 1b; Figures S3 and S4, Supporting Information). CP particles, initially 6.73 ± 1.91 nm in diameter, transformed into smaller Cu/CP nanocomposites with a size of 4.09 ± 1.23 nm during the discharge process, as CP partially transformed into Cu and soluble Li_4_P_2_O_7_, with the latter removed by water (Figure S5, Supporting Information). Elemental mapping analysis, X‐ray photoelectron spectroscopy (XPS), Auger spectroscopy, and infrared spectroscopy further verified the presence of Cu, P, and O elements in the catalysts, as well as their respective oxidation states (Figure 1c; Figure S6, Supporting Information). Notably, distinct Cu‐rich nanoparticles were observed, with uniformly distributed O and P elements. These findings consistently demonstrate the successful formation of Cu/CP nanocomposites through the Li‐ion battery discharge process.

### 2.2 Methanol Production by Electrochemical CO2RR in H‐Cells

We conducted electrochemical CO2RR experiments using Cu foils coated with pristine CP or discharged CP. The CO2RR experiments were performed in H‐cells with 0.1 m KHCO_3_ or 0.1 m CsHCO_3_ electrolytes for 1 h (Figure 1d). For all pristine and discharged CP samples, the current density increased as the applied potential became more negative. In general, discharged CP samples exhibited higher current densities than pristine CP. However, when analyzing the current density profile at −1.2 V versus RHE, we found that the current density gradually decreased as the CP sample was discharged from CP‐1.5 to CP‐0.8 (Figures S7–S11 and Table S3, Supporting Information). Notably, all tested samples produced methanol, with CP discharged at lower voltages showing higher Faradaic efficiencies for methanol. While pristine CP achieved a Faradaic efficiency of 23.4% in 0.1 m KHCO_3_ at ‐1.2 V versus reversible hydrogen electrode (RHE), CP‐0.8 reached 48.8% under the same conditions. Using 0.1 m CsHCO_3_, known to enhance CO_2_ adsorption and improve CO2RR selectivity^[^ ^25^ ^]^ further increased the Faradaic efficiency for methanol to 70.1% at ‐1.1 V versus RHE (Figure 1d; Figure S12 and Table S4, Supporting Information). Interestingly, CP‐0.8 also produces a small amount of formic acid, which decreased as the cathodic potential and methanol production increased, suggesting methanol production via the formic acid pathway (Figure S13, Supporting Information). Due to its high selectivity, CP‐0.8 demonstrated an exceptionally high methanol partial current density, exceeding −5 to −10 mA cm^−2^ in H‐cells (Figure 1e; Figure S14, Supporting Information). Additionally, CP‐0.8 maintained a Faradaic efficiency greater than 50.0% at −1.2 V versus RHE in 0.1 m CsHCO_3_ for >12 h (Figure 1f; Figure S15 and Tables S5 and S6, Supporting Information). It is worth noting that the observed gradual decrease in current density may be attributed to the depletion of dissolved CO_2_ in the H‐cells. Indeed, refreshing the electrolyte and intermittently purging it with CO_2_ enabled sustained methanol production, confirming the robust catalytic performance of CP‐0.8 (Figure S16 and Table S7, Supporting Information).

### 2.3 Active Site Identification by Ex Situ and In Situ Analyses

**Figure 2:**
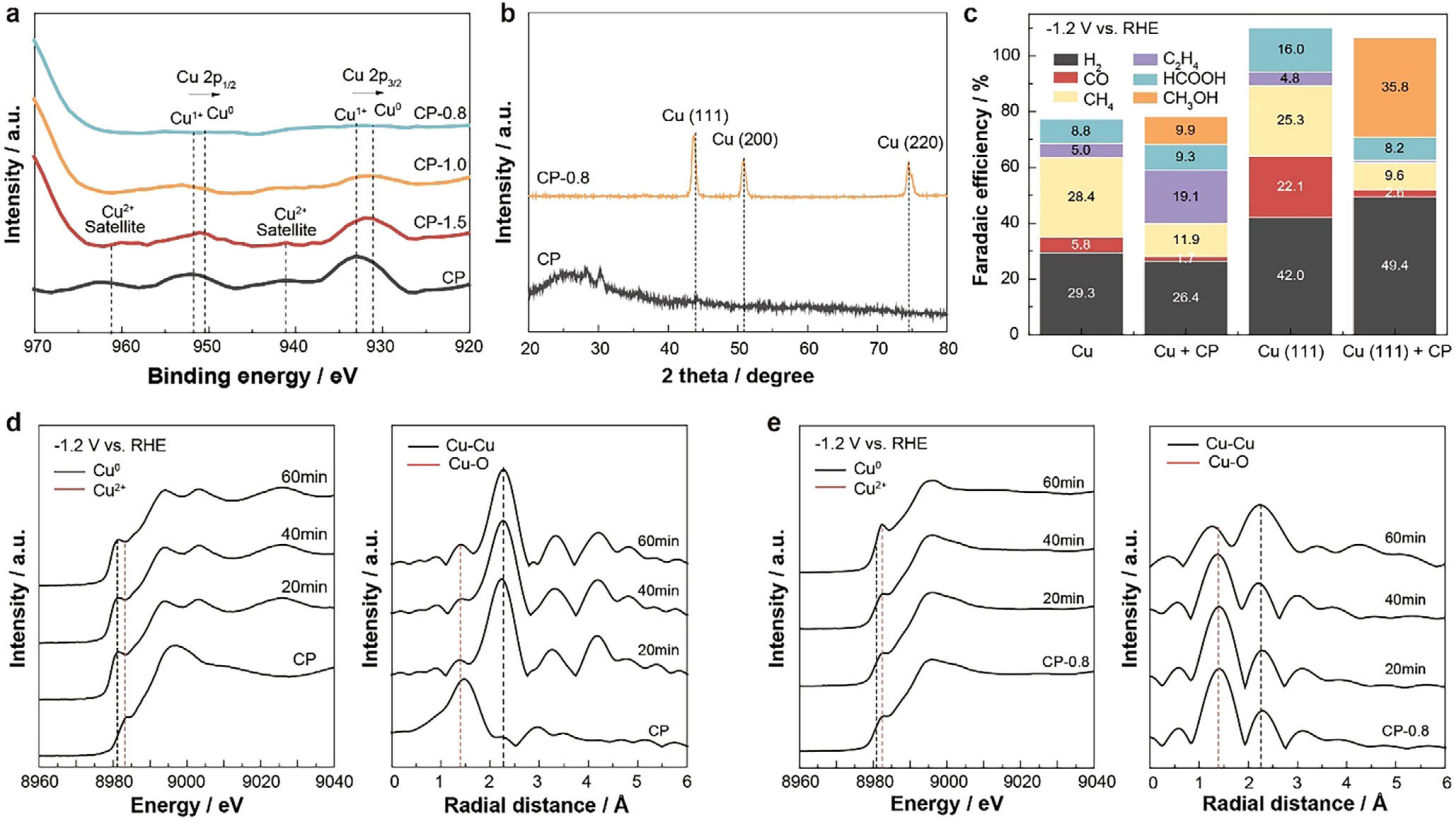
Ex situ and in situ analyses of the catalyst. a) XPS spectra of pristine CP and discharged CP catalysts. b) XRD patterns of pristine CP and CP‐0.8. c) Faradaic efficiencies of polycrystalline Cu and single‐crystalline Cu (111) with and without pristine CP modification. d,e) In situ X‐ray absorption fine structure spectra of pristine CP (d) and CP‐0.8 (e) in 0.1 m KHCO_3_ at −1.2 V versus RHE.

To identify the active sites of CP‐0.8 for selective methanol production, we conducted various ex situ analyses, including XPS and X‐ray diffraction (XRD). XPS analysis revealed multiple Cu oxidation states in both pristine and discharged CP samples: Cu 2p_1/2_ at 951–953 eV and Cu 2p_2/3_ at 932–934 eV, corresponding to Cu^+^ and Cu^0^ as well as Cu satellite peaks at 940–945 and 960–965 eV, indicative of Cu^2+^ (**Figure** **2**a). With further discharging to 0.8 V, more Cu^+^ was reduced to metallic Cu, accompanied by the disappearance of Cu^2+^ and a decrease in P content (Figure S6b and Table S8, Supporting Information). XRD analysis showed a prominent Cu (111) peak in CP‐0.8, whereas pristine CP showed no distinct peaks (Figure 2b). Based on these findings, we hypothesized that the coexistence of Cu^2+^ and/or Cu^1+^ in CP and metallic Cu^0^ in Cu (111) creates a synergistic effect, enhancing methanol selectivity.

To test this hypothesis, we conducted CO2RR at −1.2 V versus RHE using polycrystalline Cu and single‐crystalline Cu (111) foils prepared via contact‐free annealing,^[^ ^26^ ^]^ both with and without pristine CP (Figure 2c; Figure S17 and Table S9, Supporting Information). Polycrystalline Cu or single‐crystalline Cu (111) alone primarily produced methane, along with carbon monoxide, formic acid, and ethylene, as previously reported^[^ ^27^ ^]^ but no methanol. Interestingly, single‐crystalline Cu (111) foil modified with CP produced methanol with a Faradaic efficiency of 35.8%, albeit lower than the 48.8% achieved by CP‐0.8, which contains nanoparticulate Cu (111) and CP. Modifying polycrystalline Cu with CP also produced methanol, but at a lower selectivity, likely due to the presence of Cu (111) planes. These results suggest a synergistic effect between Cu (111) and CP at the nanoscale, enabling highly selective methanol production.

Given the dynamic changes often observed in Cu‐based catalysts during CO2RR^[^ ^28^ ^]^ and their associated stability challenges,^[^ ^29^ ^]^ we characterized pristine CP and CP‐0.8 via in situ X‐ray absorption spectroscopy to confirm active sites and stability. For pristine CP, oxidized Cu states were rapidly reduced to metallic Cu within the first 20 min, accompanied by bond length changes reflecting the conversion of most Cu─O bonds to Cu─Cu bonds (Figure 2d). Interestingly, a Cu (111) peak also appeared in pristine CP after CO2RR, potentially explaining the small amount of methanol produced by pristine CP (Figure S18, Supporting Information). In contrast, CP‐0.8 maintained its Cu oxidation state and Cu‐O bonds throughout the CO2RR, after an initial slight reduction of oxidized Cu to metallic Cu (Figure 2e). These findings underscore the significance of tailoring the Cu and CP composition for enhanced stability and suggest that the optimal CP content in CP‐0.8 supports improved stability, as discussed further in a subsequent section. Notably, this enhanced stability is also attributed to the stronger binding of P_2_O_7_^4−^ to transition metals compared to O^2−^. (e.g., in CuO and Cu_2_O, which are commonly used in CO2RR but suffer from poor stability).^[^ ^30^ ^]^

### 2.4 Experimental and Theoretical Verification of the Reaction Pathway for Methanol Production

**Figure 3:**
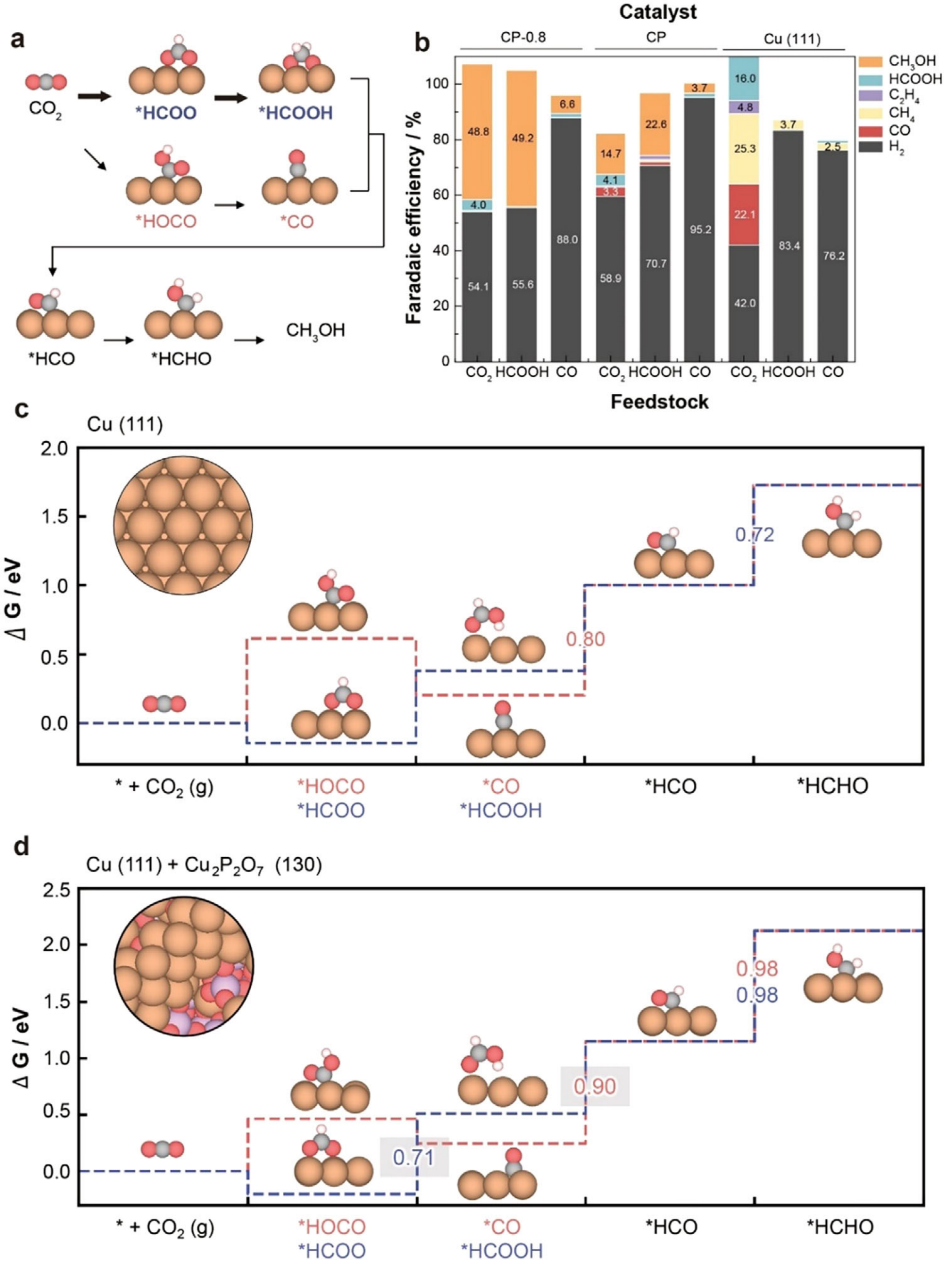
Experimental and computational investigation of the reaction pathways. a) Schematic illustration of pathways to form *HCHO intermediates crucial for methanol production via CO2RR. b) Faradaic efficiency of CP‐0.8 at −1.2 V versus RHE in a 0.1 m KHCO_3_ electrolyte with different feedstocks: CO_2_, HCOOH, and CO. c,d) DFT‐calculated Gibbs energy diagrams for the CO pathway (red line) and HCOOH pathway (blue line) on Cu (111) (c) and Cu (111) + Cu_2_P_2_O_7_ (130) (d) surfaces. The Gibbs energy values of the rate‐determining step are reported within the figure, with the second highest energy values shaded in grey shadow. Cu, O, C, H, and P atoms are represented by orange, red, grey, white, and purplish‐grey, respectively.

To elucidate the reaction pathway for efficient methanol production, we conducted electrochemical reactions using various feedstocks, including CO_2_, CO, and HCOOH. According to the literature,^[^ ^31^ ^]^ formaldehyde (^*^HCHO) is a key intermediate in methanol production via CO2RR and can be formed through two distinct pathways: the ^*^CO pathway and the ^*^HCOOH pathway (**Figure** **3**a). For CP‐0.8 catalysts, significant amounts of methanol were produced when CO_2_ and formic acid were used as feedstocks, whereas only negligible methanol was generated when CO was used as the feedstock (Figure 3b; Figure S19 and Table S10, Supporting Information). Interestingly, methanol production from CO_2_ and formic acid was also observed when using pristine CP as a catalyst. In contrast, Cu (111) alone produced no methanol, regardless of the feedstock type, although a small amount of formic acid was generated when using CO_2_. These findings suggest a synergistic effect between Cu (111) and CP in CP‐0.8, with Cu (111) responsible for formic acid formation as an intermediate and CP promoting its subsequent conversion to methanol. This aligns with our observation that discharged CP produces HCOOH but not CO at low applied potentials (Figure S13, Supporting Information). This result is noteworthy, as conventional studies have predominantly emphasized the ^*^CO pathway for methanol production.^[^ ^32^ ^]^

To understand the structure of discharged CP and the catalytic mechanism at the atomic scale, we performed DFT calculations to elucidate their surface structures and associated physicochemical properties. For modeling the Cu (111) + Cu_2_P_2_O_7_ (130) interface, consistent with high‐resolution TEM and fast Fourier transform analysis (Figure 1b; Figures S4 and S5, Supporting Information), we used the *Cellmatch* code^[^ ^33^ ^]^ to align Cu_2_P_2_O_7_ (130) with Cu (100), achieving a strain rate of less than 2%. We then constructed the Cu (111) + Cu_2_P_2_O_7_ (130) interface by introducing additional Cu atoms (Figure S20a, Supporting Information). After structure optimization, we found that the bottom layers of the Cu (111) surface were mildly reconstructed/oxidized (Figure S20b,c, Supporting Information). Radial distribution function analysis revealed that the main peak of the reconstructed Cu (111) surface was broadened compared to the ideal bulk‐truncated surface, and the first peak position shifted slightly lower (Figure S21, Supporting Information). We did not consider Cu_2_P_2_O_7_ (130) as an active site, because it exhibited significantly higher Gibbs energy (0.42–1.15 eV) for CO adsorption, compared to Cu (111) (−0.41 eV), and in some instances, CO was detached from the surface upon relaxation (Figure S22, Supporting Information).

Next, we investigated the catalytic mechanism on both clean Cu (111) (Figure 3c) and Cu (111) + Cu_2_P_2_O_7_ (130) slabs (Figure 3d) to better understand the role of Cu_2_P_2_O_7_ in the catalytic reaction. According to the literature^[^ ^8^, ^34^ ^]^ the CH_2_O_2_ adsorbs in several geometrical configurations, specifically as H_2_COO, HCOOH‐1, and HCOOH‐2. Using these configurations, we found that their total energy differences fall within 6–8 meV atom^−1^. Since experimentally identifying the preferred adsorption geometry is challenging, our computational model included all three binding geometries, and we report the configuration with the lowest Gibbs free energy for the potential‐determining step (PDS). Results for other geometries are presented in Figure S23 (Supporting Information).

On a clean Cu (111) surface, the rate‐determining step for the CO pathway was ^*^CO → ^*^HCO, with a Gibbs energy difference of 0.80 eV. For the CH_2_O_2_ pathway, the rate‐determining step was either ^*^HCOO → ^*^HCOOH or ^*^HCO → ^*^HCHO, with Gibbs energy differences ranging from 0.72 to 0.94 eV (Figure 3c; Figure S23a, Supporting Information). The Gibbs energy differences for the PDS in the HCOOH pathway were within 0.1 eV of the CO pathway, while the H_2_COO pathway exhibited a higher energy difference of ≈0.14 eV compared to the CO pathway. This suggests that the catalytic reactions involving CO are energetically competitive on clean Cu (111), aligning with experimental results (Figure 2c).

In contrast, on the Cu (111) + Cu_2_P_2_O_7_ (130) slab, the rate‐determining steps for the CO and CH_2_O_2_ pathways are the same, namely ^*^HCO → ^*^HCHO, except for the H_2_COO path. The H_2_COO pathway was excluded due to its high Gibbs free energy barrier (1.92 eV), indicating that the reaction is unlikely to proceed via this route (Figure S23b, Supporting Information). Since the CO and other CH_2_O_2_ pathways share the same rate‐determining steps, we investigated the second‐highest energy steps. For the CO pathway, the second‐highest energy step is ^*^CO → ^*^HCO, with a Gibbs energy of 0.90 eV. In the case of the HCOOH‐1 pathway, the Gibbs energy (0.83 eV) is comparable to that of the CO pathway; however, the HCOOH‐2 pathway shows a significantly lower Gibbs energy, with a difference of ≈0.2 eV (Figure 3d). In the Cu (111) + Cu_2_P_2_O_7_ (130) configuration, the CO pathway now requires a higher energy barrier, which explains why the Faradaic efficiency for methanol under a CO atmosphere is noticeably low (Figure 3b).

For a simplified model, we also investigated Cu (111) + PO_4_. First, we assessed the relative thermodynamic stability as a function of the number of PO_4_ adsorptions and pH conditions (Figures S24–S26, Supporting Information). We created surface slab models with 1–4 PO_4_ molecules on (4 × 4) Cu (111) slabs and calculated the Gibbs free energy of adsorption (Δ*G*_2_) under varying pH conditions, following previous studies.^[^ ^35^ ^]^ Further details on the calculations are provided in the Supplementary Information. Our calculation revealed that the Cu (111) surface with a small amount of PO_4_ is the most thermodynamically stable. This finding supports the superior long‐term stability of CP‐0.8 (Figure 2e), which contains only small amounts of PO_4_. On Cu (111) + PO_4_, there is a clear preference for the CH_2_O_2_ pathway over the CO pathway. All CH_2_O_2_‐related pathways exhibited lower Gibbs energies for the PDS compared to the CO pathway, with absolute differences exceeding 0.1 eV (Figure S22c, Supporting Information). Notably, in the H_2_COO and HCOOH‐1 cases, the energy differences were greater than 0.2 eV. These findings further support the critical roles that both the Cu (111) surface and phosphate‐related species play in altering the reaction pathway.

To further confirm the proposed reaction pathway, *operando* Raman spectroscopy was conducted to identify key intermediates (Figure S27, Supporting Information). However, distinguishing the characteristic Raman peak for formate species (≈1300–1400 cm^−1^) was challenging due to interference from the D‐band of carbon incorporated during the catalyst synthesis (≈1350 cm^−1^).^[^ ^36^ ^]^ Nevertheless, in situ IR spectroscopy clearly identified the adsorbed formate (^*^HCOO) species, showing distinct characteristic peaks ≈1390 and 1280 cm^−1^, along with an additional shoulder peak at ≈1570 cm^−1^ (Figure S28, Supporting Information).^[^ ^37^ ^]^ The formate species rapidly decreased at higher potentials, indicating the consumption of formate species for facilitating methanol production. These IR results highly support the formation and adsorption of formate intermediate on the catalyst surface, emphasizing the validity of the proposed HCOOH reaction pathway.

### 2.5 Methanol Production by CO2RR Under Various pH Conditions and in GDE Cells

**Figure 4:**
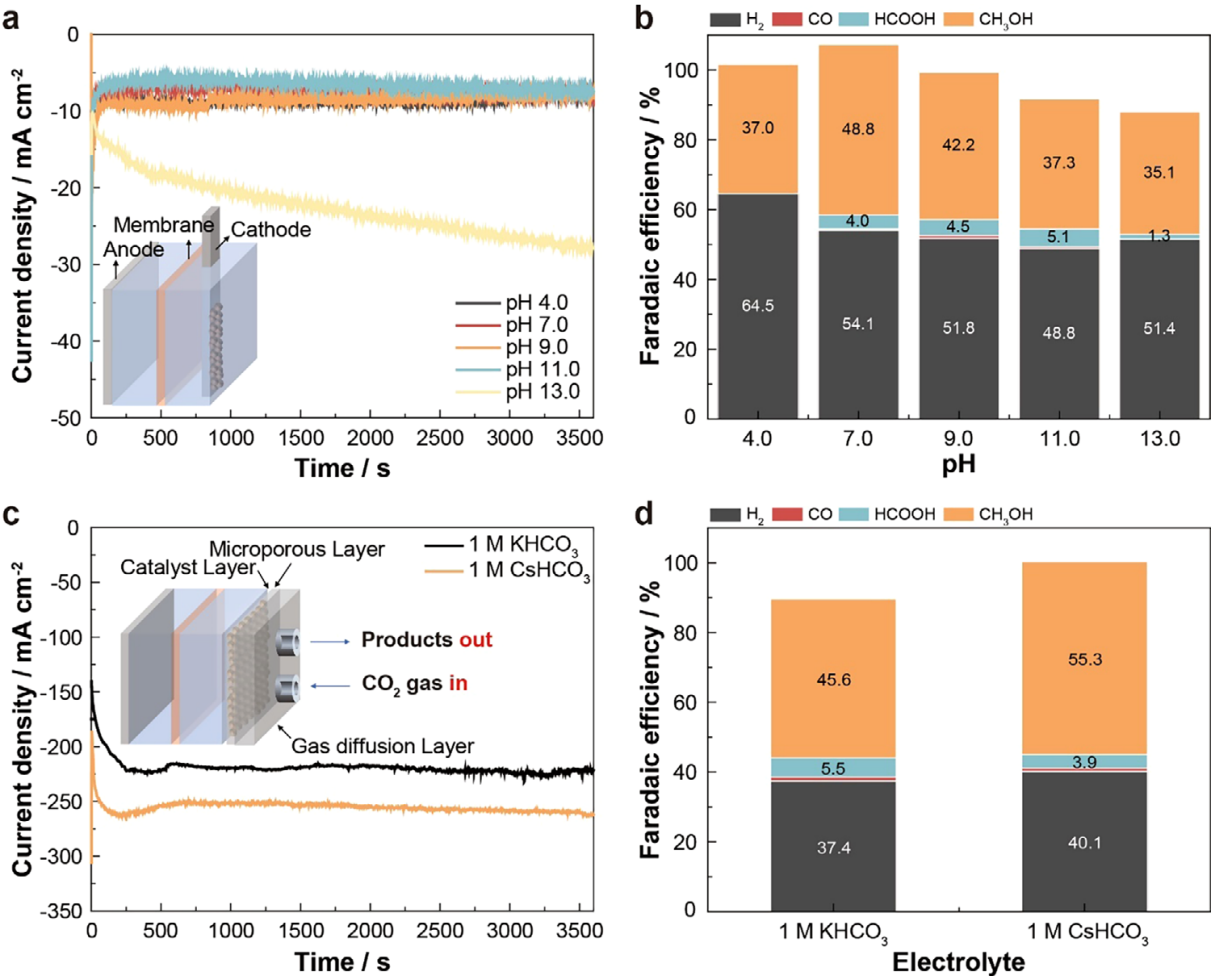
Electrochemical performance under various pH conditions and in a flow cell. a, b) Current density (a) and Faradaic efficiency (b) of CP‐0.8 under different pH conditions (Inset: schematic of the H‐cell setup). c,d) Current density (c) and Faradaic efficiency (d) of CP‐0.8 at −1.0 V versus RHE in 1 m KHCO_3_ and 1 m CsHCO_3_ electrolytes (Inset: schematic of the GDE setup).

To evaluate methanol selectivity under different pH conditions, we prepared electrolytes with pH values of 4.0, 7.0, 9.0, 11.0, and 13.0 (**Figure** **4**a,b; Table S11, Supporting Information). While pristine CP predominantly produced hydrogen across these pH conditions, CP‐0.8 consistently showed high methanol selectivity, with Faradaic efficiencies exceeding 30% (Figure S29, Supporting Information). In particular, CP exhibited the highest Faradaic efficiency for methanol near neutral pH, likely due to increased CO_2_ availability (not present in the form of carbonate) and reduced competition from C_2_ product pathways, as previously reported.^[^ ^38^ ^]^ The pH dependence of methanol production showed a similar trend regardless of the feedstock─CO_2_, HCOOH, or CO (Figure S30, Supporting Information). These results highlight the superior catalytic activity and stability of CP‐0.8 for selective methanol production in CO2RR across a wide pH range, suggesting a consistent reaction pathway. Nevertheless, further studies are required to fully validate these findings. Notably, CP‐0.8 displayed peak activity near neutral pH, close to its pKa value, suggesting that an optimal degree of protonation and/or adequate ^*^H coverage are critical for efficient methanol production.

To further enhance the methanol production rate, we employed a GDE in a flow cell setup. At −1.0 V versus RHE, the GDE cells using CP‐0.8 achieved methanol Faradaic efficiency of 45.6% in 1 m KHCO_3_ and 65.7% in 1 m CsHCO_3_ (Figure 4c,d; Figures S31 and S32 and Tables S12 and S13, Supporting Information). The partial current density for methanol production with CP‐0.8 in GDEs reached 113.3 mA cm^−2^ in 1 m KHCO_3_ and 145.5 mA cm^−2^ in 1 m CsHCO_3_, significantly surpassing the performance of pristine CP in GDE cells (23.9 mA cm^−2^) and CP‐0.8 in H‐cells (Figure S31, Supporting Information). To the best of our knowledge, a methanol partial current density exceeding 100 mA cm^−2^ represents one of the highest values reported to date for catalysts without noble metals and the highest among Cu‐based catalysts in this category. In particular, CP‐0.8 showed the best Faradaic efficiency for methanol of 65.7% at −0.8 V versus RHE (Figure S33 and Tables S14–S16, Supporting Information).

The Cu/Cu_2_P_2_O_7_ (Cu/CP)‐based hybrid catalyst represents a significant breakthrough in CO_2_ reduction reaction for methanol production. This catalyst not only achieves one of the highest methanol partial current densities reported for Cu‐based electrocatalysts, exceeding 100 mA cm^−2^, but also does so with remarkable efficiency, making it stand out among non‐noble metal‐based catalysts (Tables S15 and S16, Supporting Information). Its cost‐effectiveness and scalability further enhance its potential, as the Cu/CP catalyst operates efficiently in aqueous electrolytes with high Faradaic efficiency. The catalyst's performance in gas diffusion electrode (GDE) systems offers additional advantages, making it a promising candidate for large‐scale industrial applications. This advancement holds significant implications for CO2RR research, as the Cu/CP catalyst offers a sustainable, high‐performance solution for methanol synthesis. Methanol, a highly valuable renewable fuel and versatile chemical feedstock, plays a crucial role in producing industrial chemicals, including aldehydes, olefins, and resins. With its exceptional efficiency, cost‐effectiveness, and compatibility with GDE systems, the Cu/CP catalyst is poised to contribute significantly to the industrial adoption of CO_2_ reduction technologies, particularly in the fields of renewable fuels, chemical manufacturing, and energy storage.

Nevertheless, there are still some limitations that require further investigation for practical application. For example, despite significant progress, the selectivity and current density for methanol production remain lower than those achieved for other CO2RR products such as CO, formic acid, and C_2_H_4_, which exhibit Faradaic efficiencies approaching or even exceeding 70–80% and partial current densities ranging from several hundred mA cm^−2^ to over 1 A cm^−2^. Catalyst stability must also be evaluated and enhanced beyond the laboratory scale (Figure S16, Supporting Information). Additionally, the current synthesis method relies on battery discharge cycles, which limits scalability. Therefore, alternative synthesis approaches based on the design principles proposed in this study should be further explored to enable large‐scale application.

## 3 Conclusion

In summary, we synthesized Cu/CP‐based hybrid catalysts for efficient and selective methanol production via CO2RR. These hybrid catalysts were tailored through a discharge process of Li‐ion batteries, with the CP‐0.8 catalyst demonstrating the highest efficiency and stability for methanol production. Ex situ analysis revealed that the superior catalytic activity of CP‐0.8 arises from a synergistic effect between metallic Cu nanoparticles with a predominant (111) surface and CP nanoparticles, which facilitate formic acid formation as an intermediate and its subsequent conversion to methanol, respectively. In situ X‐ray absorption spectroscopy further confirmed the stable presence of both metallic and oxidized Cu species in CP‐0.8 during CO2RR. Electrochemical reactions using various feedstocks, supported by DFT calculations, demonstrated that methanol production proceeds via the HCOOH pathway on Cu (111) in the presence of phosphate. CP‐0.8 catalysts exhibited a high Faradaic efficiency of 50–70% and one of the highest reported methanol partial current densities exceeding 100 mA cm^−2^ in GDE cells. These findings provide valuable insights into the design and synthesis of novel CO2RR electrocatalysts, offering a promising pathway for efficient and sustainable methanol production.

## Conflict of Interest

The authors declare no conflict of interest.

## Supporting information



Supporting Information

## Data Availability

The data that support the findings of this study are available from the corresponding author upon reasonable request.
