# Supplementary material for: Selective Electrosynthesis of Methanol from CO2 Over Cu/Cu2P2O7 Via the Formate Pathway
Source: Adv Mater. 2025 May 20;37(35):2501021. doi: 10.1002/adma.202501021 (PMC12411997; doi:10.1002/adma.202501021)
Supplement: Supplementary file 1 — Supporting Information [file ADMA-37-2501021-s001.pdf]

ADVANCED MATERIALS

Supporting Information

for *Adv. Mater.*, DOI 10.1002/adma.202501021

Selective Electrosynthesis of Methanol from CO_2 Over $Cu/Cu_2P_2O_7$ Via the Formate Pathway

Hyunwoo Kim, Jihoe Lee, Sangseob Lee, Suhwan Park, Yongseok Lee, Giyeok Lee, Hyo Sang Jeon, Man Ho Han, Sunghwan Jin, Hyun-Wook Lee, Aloysius Soon, Jongsoon Kim* and Jungki Ryu**

Supporting Information

Selective Electrosynthesis of Methanol from CO_2 over $Cu/Cu_2P_2O_7$ via the Formate Pathway

Hyunwoo Kim, Jihoe Lee, Sangseob Lee, Suhwan Park, Yongseok Lee, Giyeok Lee, Hyo Sang Jeon, Man Ho Han, Sunghwan Jin, Hyun-Wook Lee, Aloysius Soon, Jongsoon Kim*, and Jungki Ryu**

H. Kim, S. Park, H.-W. Lee, J. Ryu
School of Energy and Chemical Engineering
Ulsan National Institute of Science and Technology (UNIST)
Ulsan 44919, Republic of Korea
E-mail : jryu@unist.ac.kr

J. Lee, Y. Lee, J. Kim
Department of Energy Science
Sungkyunkwan University (SKKU)
Suwon 16419, Republic of Korea
E-mail : jongsoonkim@skku.edu

S. Lee, G. Lee, A. Soon
Department of Materials Science and Engineering
Yonsei University
Seoul 03722, Republic of Korea
E-mail : Aloysius.soon@yonsei.ac.kr

H. S. Jeon
Sustainable Energy Research Division
Korea Institute of Science and Technology (KIST)
Seoul 02792, Republic of Korea

M. H. Han
Clean Energy Research Center
Korea Institute of Science and Technology (KIST)
Seoul 02792, Republic of Korea

S. Jin
Department of Materials Science and Engineering
Kangwon National University
Samcheok 25913, Republic of Korea

J. Kim
SKKU Institute of Energy Science and Technology (SIEST)
Sungkyunkwan University (SKKU)

Suwon 16419, Republic of Korea

J. Ryu
Graduate School of Carbon Neutrality
Ulsan National Institute of Science and Technology (UNIST)
Ulsan 44919, Republic of Korea

J. Ryu
Center for Renewable Carbon
Ulsan National Institute of Science and Technology (UNIST)
Ulsan 44919, Republic of Korea

Experimental Section

Synthesis of $Cu_2P_2O_7$ (CP) composites

To prepare crystalline CP powders, CuO (99%) and $NH_4H_2(PO_4)$ (98%) precursors were mixed via wet ball milling at 300 rpm for 12 h in acetone. The resulting mixture was dried at 80 °C for 12 h with stirring on a hot plate, then ground into a fine powder, and then calcined at 850 °C for 10 h in air. The synthesized CP exhibited a light emerald color. High-energy ball milling was then used to mix the pristine CP powder with conductive carbon. Specifically, 80 wt% CP and 19 wt% Super P carbon black were placed in a nitride jar with 30 balls and ball-milled at 500 rpm for 15 h. Finally, 1 wt% carbon nanotubes (CNTs) were incorporated with the CP by high-energy ball milling at 100 rpm for 12 h.

Synthesis of CP electrode

To fabricate the CP electrodes, a slurry was prepared containing 87.5 wt% CP composite, 2.5 wt% multi-wall carbon nanotubes (MWCNTs), and 10 wt% polyvinylidene fluoride (PVDF) binder in N-methyl-2-pyrrolidone, resulting in 70:20:10 ratio of CP, conductive carbon, and PVDF binder, respectively. The slurry was applied onto Cu foil with an areal mass loading of ~2 mg/cm^2 and dried in an oven at 110 °C for 12 h.

Synthesis of discharged CP electrode

CP cells were assembled in an Ar-filled glove box using R2032 cell-type components, including the CP electrode, a Li counter electrode, a separator (Celgard, USA), and an electrolyte composed of 1.2M $LiPF_6$ in 2:4:4 (v/v) mixture of ethylene carbonate (EC), ethyl methyl carbonate (EMC), and dimethyl carbonate (DMC). The CP cells were discharged to cut-off voltages of 1.5, 1.2, 1.0 and 0.8 V at 30 °C using an automatic WBCS 3000 battery

charge/discharge test system (WonATech, Korea). After reaching each cut-off voltage, the cells were disassembled in an Ar-filled glove box and dried for 1 h.

Synthesis of Cu and Cu (111) foil modified with CP

For the preparation of Cu and Cu (111) substrates modified with CP, the catalyst ink was prepared by dispersing the CP powder in a liquid mixture composed of 3 mL isopropyl alcohol (IPA) and 50 μL Nation solution. The catalyst ink was then uniformly deposited onto Cu or Cu (111) single-crystal substrates using the drop-casting method, followed by drying at ambient conditions.

Structural and elemental characterization

Morphology was characterized using an S-4800 scanning electron microscope (SEM) (Hitachi High-Technologies, Japan) and a JEM-2100F transmission electron microscope (TEM) (JEOL, Japan). Crystallinity and elemental composition were analyzed using a D/MAX25000V/PC high-power X-ray diffractometer (XRD) (Rigaku, Japan) and X-ray photoelectron spectroscopy (XPS) (Thermo Fisher Scientific, USA). Chemical bonding and functional groups were examined with an infrared (IR) spectroscopy (Agilent Technologies, USA).

Electrochemical CO_2 reduction reaction

Electrochemical CO2RR was conducted using SP-150 and SP-300 potentiostat/galvanostat (Bio-Logic Science Instruments, France). All experiments were carried out in a H-cell, consisting of a working electrode (Pristine CP and discharged CP), a counter electrode (platinum electrode), and a reference electrode (Ag/AgCl). An anion exchange membrane (AEM) was used to separate the anode and cathode compartments, each containing 0.1 M $KHCO_3$ or 0.1 M $CsHCO_3$ electrolyte. In particular, 0.1 M CH_3COOK, 0.1 M $KHCO_3$, and 0.1

M KOH electrolytes were used at pH values of 4.0, 7.0 (9.0, 11.0), and 13.0, respectively. Prior to the experiment, the electrolyte was purged with CO_2 gas for at least 30 min. Potentials were referenced to the Ag/AgCl and converted to the RHE scale using the Nernst equation ($E_{RHE} = E_{Ag/AgCl} + 0.05916 \times pH + 0.197$ V). Long-term stability was assessed for 12 h at 2 h intervals. During the reaction, CO_2 gas was purged every 2 h, and electrolyte samples were collected through a syringe each hour. All liquid products were subsequently analyzed via liquid chromatography.

Gas diffusion electrode (GDE) preparation and measurement

The GDE was prepared using a Freudenberg H23C8 electrode. Catalyst ink was made by mixing 3 mg of a catalyst with 3 mL of ethanol, and 120 μL of Nafion solution, followed by sonication for 1 h. This ink was spread onto the GDE to form the working electrode. All experiments were performed in a custom cell, consisting of a working electrode, a counter electrode (platinum wire), and a reference electrode (Ag/AgCl). Both anodic and cathodic compartments contained 1 M $KHCO_3$ or 1 M $CsHCO_3$ electrolyte, with the electrolyte flow rate set to 2 mL min^{-1} using a peristaltic pump. CO_2 flow rate was controlled at 50 mL min^{-1} using a mass flow controller.

Gas chromatography / Liquid chromatography measurement

Gas products were collected using a syringe and analyzed with a Shimadzu 2010 Plus gas chromatography (GC) (Shimadzu Co., Japan) equipped with a barrier discharge ionization detector (BID) and helium as the carrier gas. Liquid products were collected using a syringe and analyzed using a Waters 2410 high-performance liquid chromatography HPLC (Waters, USA) with 5mM H_2SO_4 as the mobile phase. The faradaic efficiency for each product was calculated using the following equation:

$$Faradaic\ efficiency\ /\ \% = \left(\frac{n \times F \times Q_{product}}{Q_{total}}\right) \times 100$$

where n is the number of electrons required per molecule of product and F is the faradaic constant. $Q_{product}$ and Q_{total} represent the charge associated with the formation of product and the total charge passed during CO2RR, respectively.

In-situ X-ray absorption spectroscopy

In-situ X-ray absorption spectroscopy (XAS) was conducted at beamline 6D of the Pohang Light Source (PLS) at the Pohang Accelerator Laboratory (PAL) in Korea. XAS spectra were acquired at the Cu K edge in fluorescence mode under the following conditions: electron energy of 3.5 GeV, a maximum current of 400 mA, and a beam size of 2 mm x 1 mm. In-situ XAS for CO2RR was carried out in $KHCO_3$ electrolyte in the custom cell. Data were analyzed using a combination of ATHENA and ARTEMIS software.

Density Functional Theory (DFT) calculations

DFT calculations were performed using the Vienna Ab initio Simulation Package (VASP) with the projector augmented wave (PAW) method.[S1] The optB86b exchange-correlation (xc) functional was selected to effectively account for the long-range van der Waals contributions in a self-consistent manner.[S2] The kinetic energy cutoff for the plane waves was set to 500 eV, with a Γ-centered k-point grid spacing of 0.15 $Å^{-1}$ and a 5 × 5 × 1 k-point grid for all surfaces. To mimic the bulk region, the bottom two layers of Cu were fixed, while the other layers were allowed to relax. To prevent unphysical interactions along the *c*-direction, a vacuum region of at least 15 Å was maintained between all slab models, and dipole correction was applied. Structure relaxations was continued until all forces were below 0.02 eV/Å. The

Gibbs energy via CO2RR was calculated based on the computational hydrogen electrode approach, using the following equation:

$$\Delta G = \Delta E + \Delta \mathrm{ZPE} + \int C_p \mathrm{d}T - T\Delta S,$$

where E, ZPE, C_p, T, and S represent the total energies from DFT, zero-point energy, heat capacity, temperature, and entropy, respectively.[S3]

Calculation process for Gibbs energy of PO_4 adsorptions on Cu (111) orientation

For calculating the PO_4 adsorption Gibbs energy (ΔG_2 in Figure S24), we follow Figure S24 scheme based on a previous study.[S4]

We consider the acid ionization constants as follows:

$$k_{\mathrm{a1}} = \frac{[\mathrm{H^+}][\mathrm{H_2PO_4^-}]}{[\mathrm{H_3PO_4}]} = 7.1 \cdot 10^{-3}$$
$$k_{\mathrm{a2}} = \frac{[\mathrm{H^+}][\mathrm{HPO_4^{2-}}]}{[\mathrm{H_2PO_4}]} = 6.2 \cdot 10^{-8}$$
$$k_{\mathrm{a3}} = \frac{[\mathrm{H^+}][\mathrm{PO_4^{3-}}]}{[\mathrm{HPO_4}]} = 4.5 \cdot 10^{-13}$$

At standard conditions, Gibbs energy (G°) of $\mathrm{H_3PO_4}$ (aq), $\mathrm{H_2PO_4^-}$ (aq), $\mathrm{HPO_4^{2-}}$ (aq), and $\mathrm{PO_4^{3-}}$(aq) are -11.842, -11.712, -11.289, and -10.558 eV, respectively.

At equilibrium conditions, Gibbs energy of $\mathrm{H_3PO_4}$, $\mathrm{H_2PO_4^-} + \mathrm{H^+}$, $\mathrm{HPO_4^{2-}} + 2\mathrm{H^+}$, and $\mathrm{PO_4^{3-}} + 3\mathrm{H^+}$ should be same. In other words, the below equation should be maintained at all pH conditions.

$$G_{\mathrm{H_3PO_4}} = G_{\mathrm{H_2PO_4^-+H^+}} = G_{\mathrm{HPO_4^{2-}+2H^+}} = G_{\mathrm{PO_4^{3-}+3H^+}}$$

In the equilibrium condition, the most protonated anion contents (x) depending on the acid ionization constant should be as follows.

$$x = \frac{k_{\mathrm{a}}}{k_{\mathrm{a}} + 10^{-\mathrm{pH}}}$$

Using k_{a1}, we can get the ΔG_1 of H_3PO_4 or $H_2PO_4^- + H^+$ as follows.

$$\Delta G_{1\_k_{a1}} = G^{\circ}_{H_3PO_4} + k_BT \cdot \ln(1 - x_1) = G^{\circ}_{H_2PO_4^-} + k_BT \cdot (\ln(x_1) + \ln 10^{-pH})$$

Same way, we can get $\Delta G_{1\_k_{a2}}$ and $\Delta G_{1\_k_{a3}}$ as follows.

$$\Delta G_{1\_k_{a2}} = G^{\circ}_{H_2PO_4^-} + k_BT \cdot (\ln(1 - x_2) + \ln 10^{-pH})$$
$$= G^{\circ}_{HPO_4^{2-}} + k_BT \cdot (\ln(x_2) + 2\ln 10^{-pH}),$$
$$\Delta G_{1\_k_{a3}} = G^{\circ}_{HPO_4^-} + k_BT \cdot (\ln(1 - x_3) + 2\ln 10^{-pH})$$
$$= G^{\circ}_{PO_4^{3-}} + k_BT \cdot (\ln(x_3) + 3\ln 10^{-pH})$$

Among $\Delta G_{1\_k_{a1}}$, $\Delta G_{1\_k_{a2}}$, and $\Delta G_{1\_k_{a3}}$, the lowest values are taken as ΔG_1.
Gibbs energy of adsorption (ΔG_2) is calculated depending on the number of PO_4 on (4 × 4) Cu (111) surface. The reaction path is set as follows.

$$* + n \cdot H_3PO_4(aq) \rightleftharpoons * n \cdot PO_4 + n \cdot (3H^+ + 3e^-)$$

Accordingly, Gibbs energy of adsorption (ΔG_2) is calculated as below.

$$\Delta G_2 = \frac{1}{n}[(E_{*n \cdot PO_4} + n \cdot \left(\frac{3}{2}E_{H_2} - 3eU - k_BT \cdot \ln 10 \cdot pH - \Delta G_1\right) - E_{Cu(111)} + \Delta ZPE - T\Delta S)]$$

$E_{*n \cdot PO_4}$, E_{H_2}, $E_{Cu(111)}$, and U represent the DFT total energy of PO_4 adsorbed on Cu(111) surface, H_2 molecule, clean Cu (111) and electrode potential, respectively.

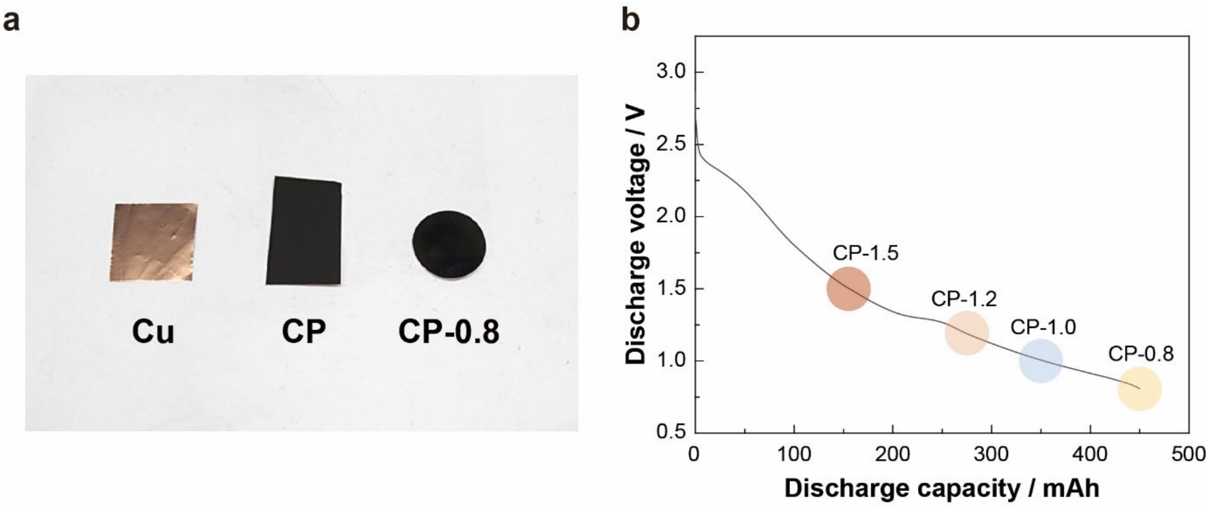

Figure S1. Digital image and CV profile of Cu/CP-based hybrid catalysts. (a) Photographs of Cu, pristine CP and CP-0.8. (b) Discharge profile of CP to prepare various Cu/CP hybrid catalysts.

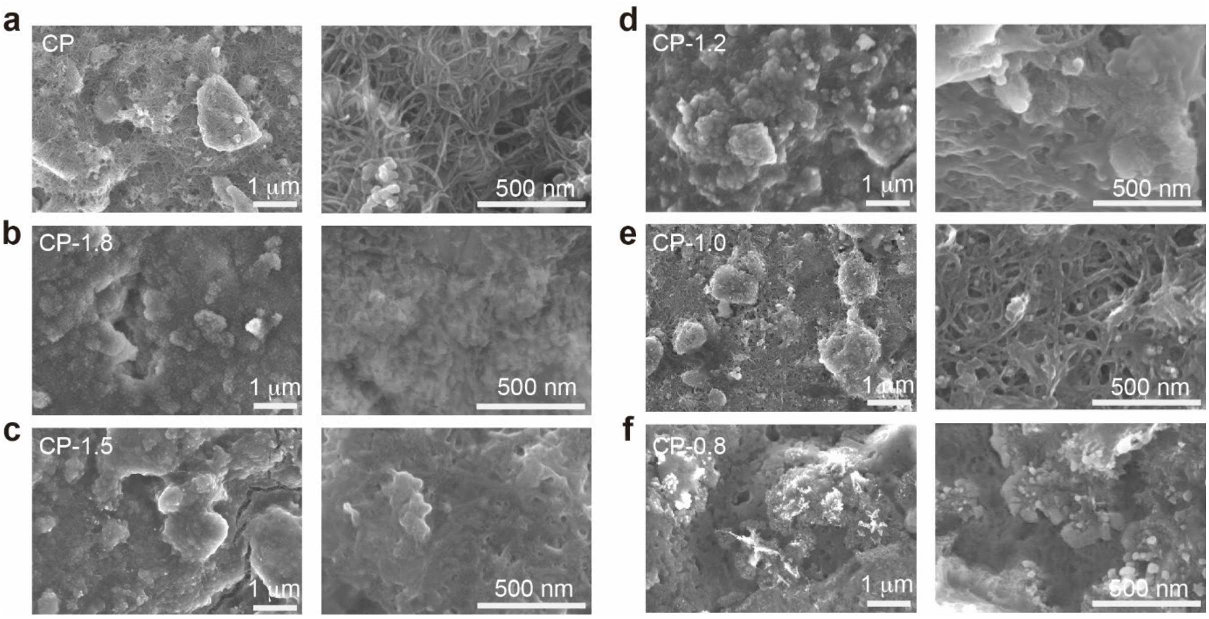

Figure S2. SEM images of pristine CP and discharged CP samples before washing with water. (a-f) SEM Images of pristine CP (a) and discharged CP (b-f). Through the conversion reaction in Li-ion batteries, a mixture of metallic Cu and $Li_4P_2O_7$ was formed, with $Li_4P_2O_7$ uniformly coating the surface.

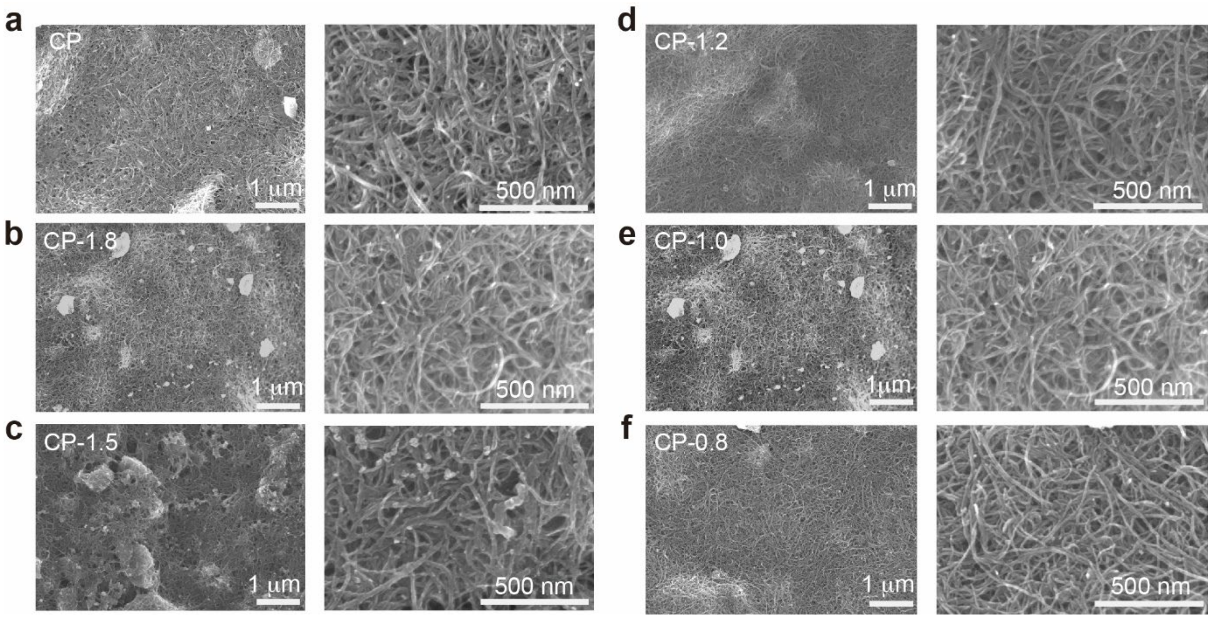

Figure S3. SEM images of pristine CP and discharged CP samples after washing with water. (a-f) Images of pristine CP (a) and discharged CP (b-f). Due to the high solubility of $Li_4P_2O_7$, pristine CP and discharge CP nanoparticles coated on CNTs were clearly observed after washing with water.

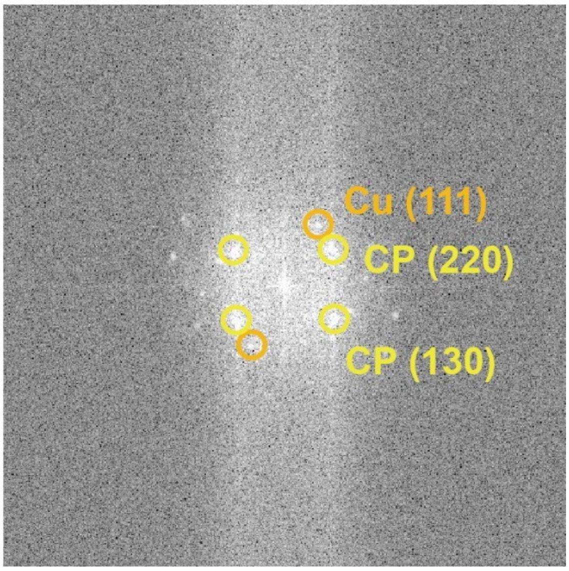

Figure S4. Fast Fourier transform (FFT) pattern derived from the TEM image of CP-0.8 (Figure 1b), showing the crystallographic orientations of Cu and CP lattice planes in CP-0.8.

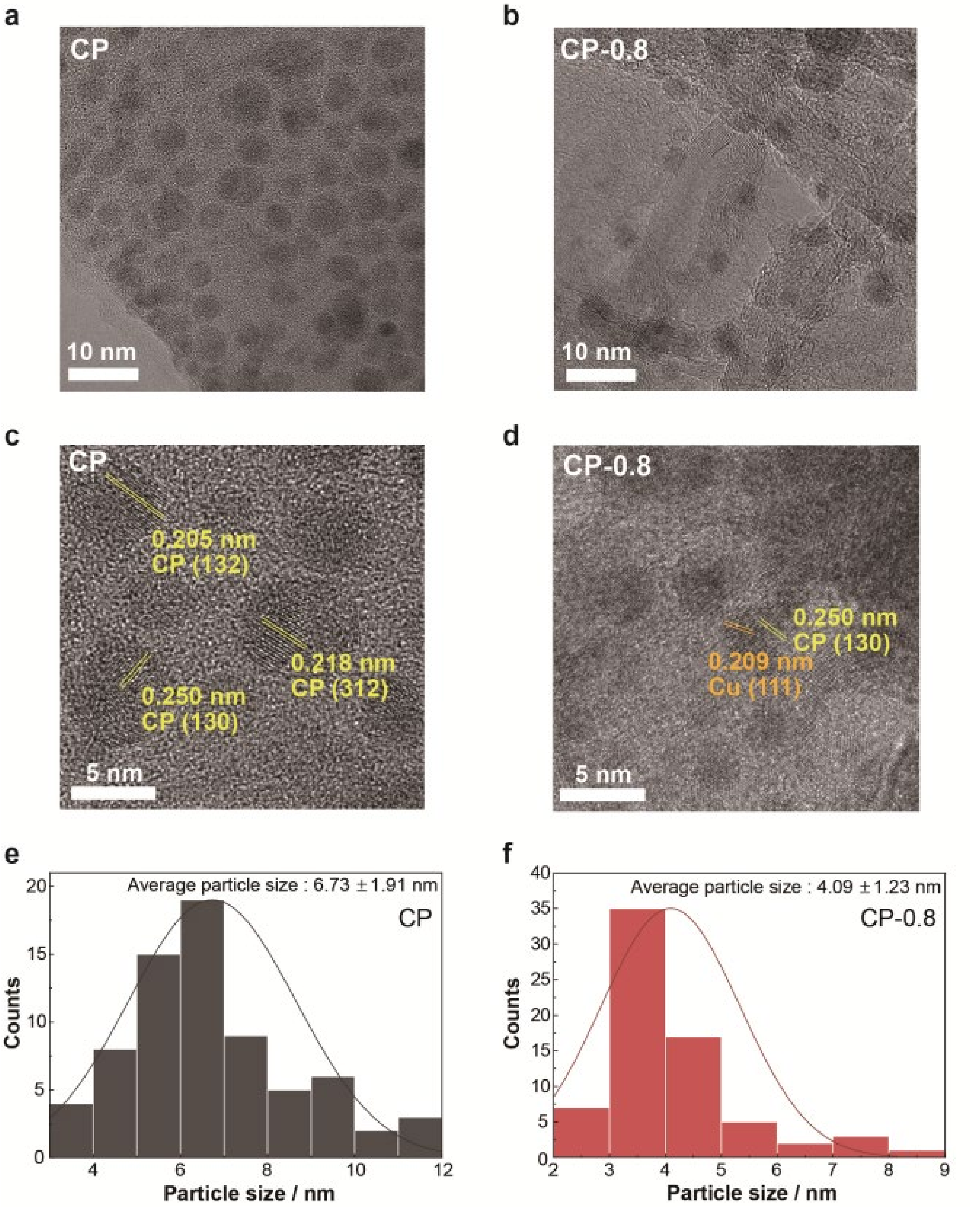

Figure S5. Characterization of CP and CP-0.8 catalysts. (a-d) HR-TEM images and (e, f) particle size distributions of CP (a, c, e) and CP-0.8 (b, d, f) catalysts.

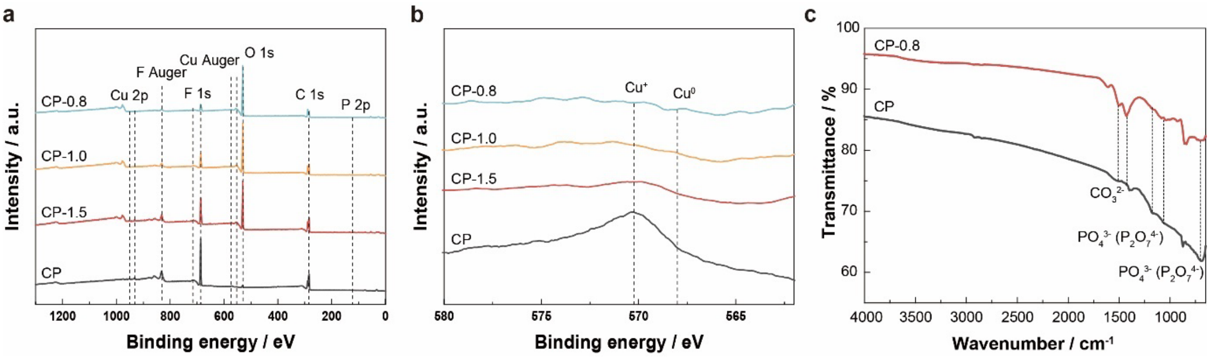

Figure S6. X-ray photoelectron spectroscopy and infrared spectroscopy of pristine CP and discharged CP. (a) XPS spectra of pristine CP and discharged CP (CP-1.5, CP-1.0 and CP-0.8), confirming the presence of Cu, O and P elements, as well as F originating from the precursors used in the Li-ion battery reaction. (b) Cu LMM Auger spectra of CP and discharged CP catalysts. (c) IR spectra showing phosphate-related peaks of pristine CP and CP-0.8.

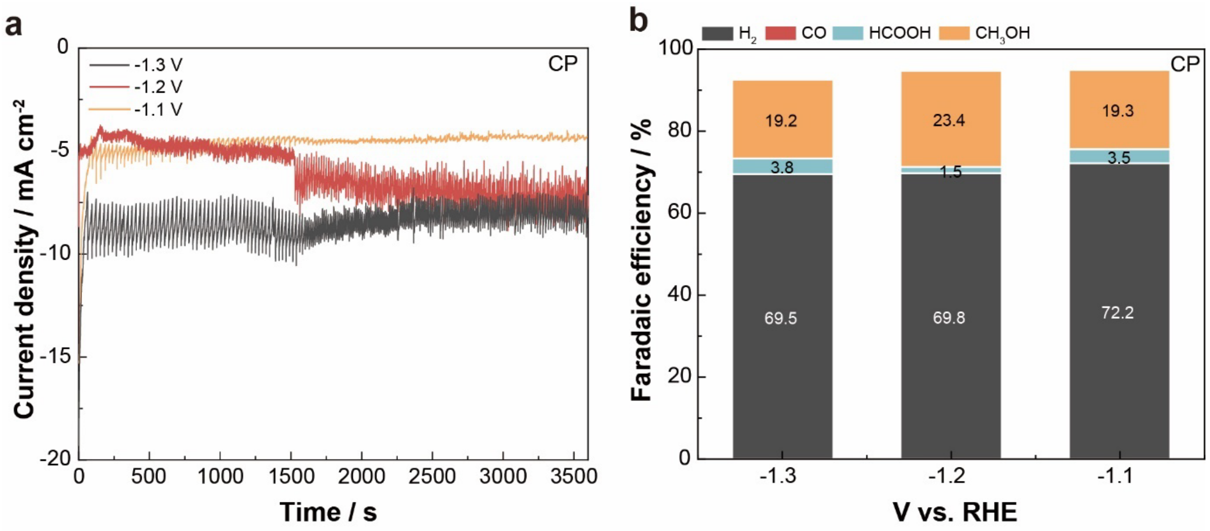

Figure S7. Electrochemical CO2RR using pristine CP in 0.1 M $KHCO_3$ electrolyte. (a) Current density profile. (b) Faradaic efficiency.

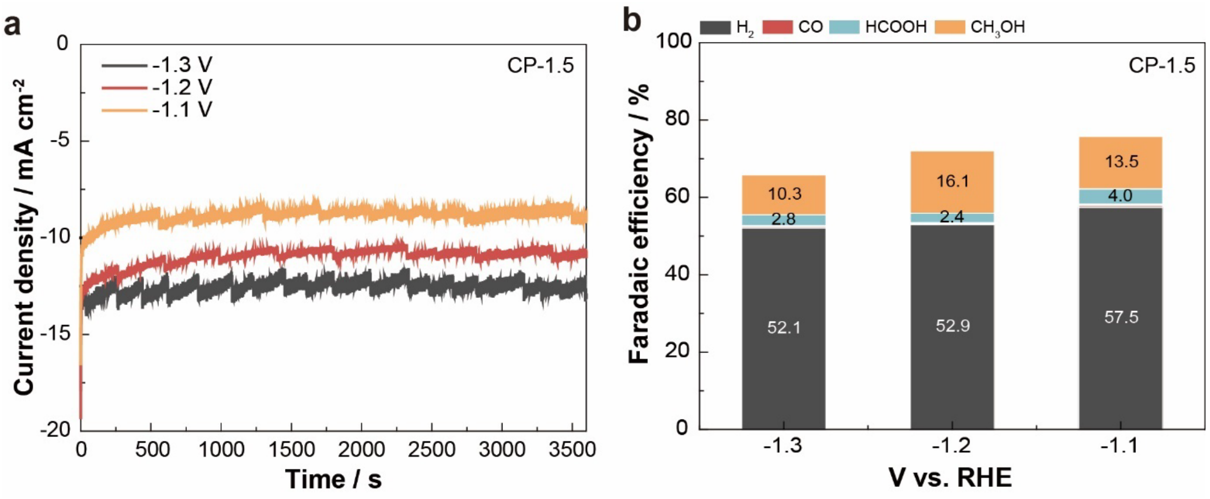

Figure S8. Electrochemical CO2RR using CP-1.5 in 0.1 M $KHCO_3$ electrolyte. (a) Current density profile. (b) Faradaic efficiency.

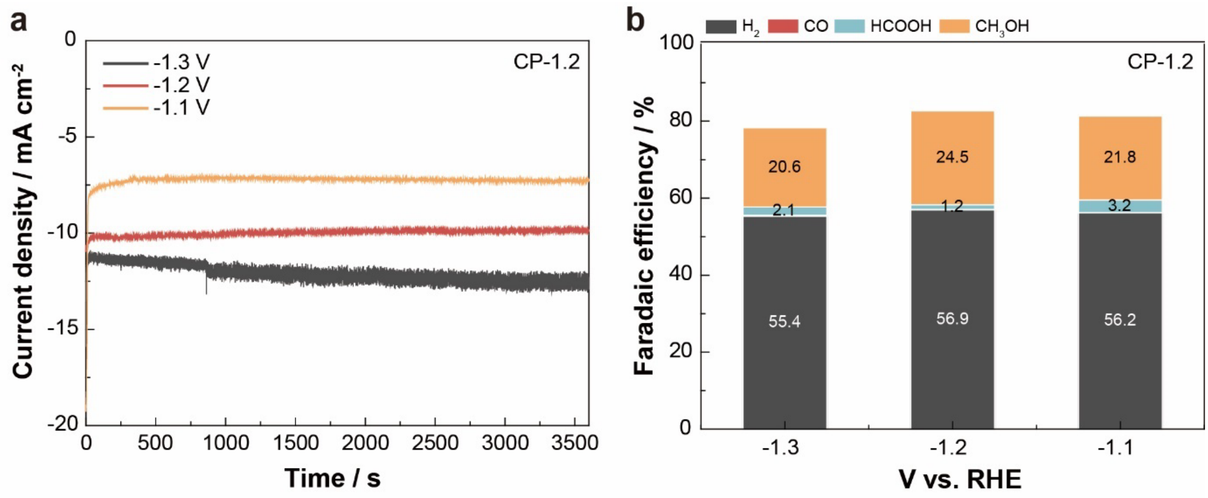

Figure S9. Electrochemical CO2RR using CP-1.2 in 0.1 M $KHCO_3$ electrolyte. (a) Current density profile. (b) Faradaic efficiency.

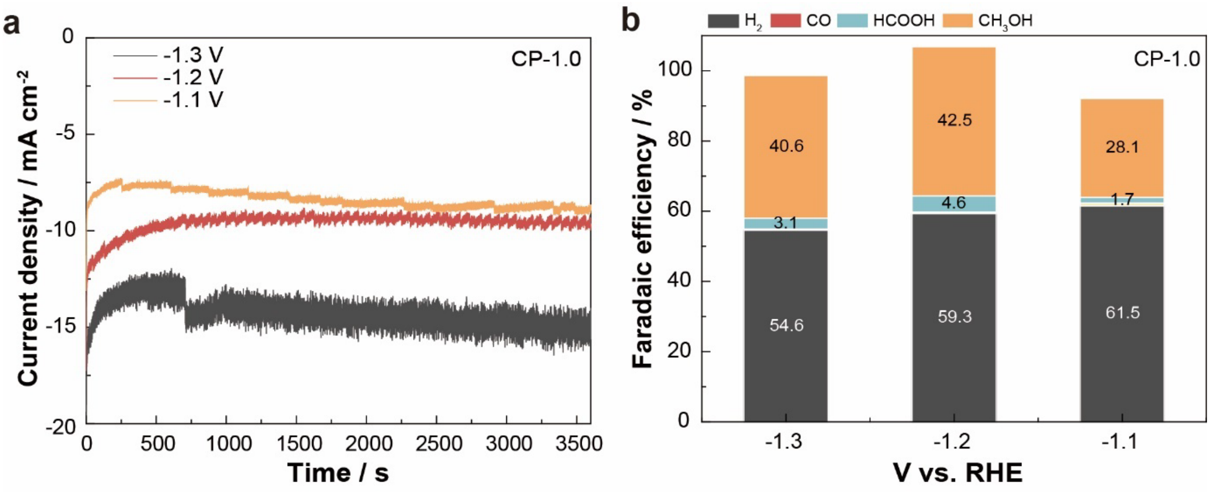

Figure S10. Electrochemical CO2RR using CP-1.0 in 0.1 M $KHCO_3$ electrolyte. (a) Current density profile. (b) Faradaic efficiency. A methanol yield of over 40.0% was achieved with CP-1.0.

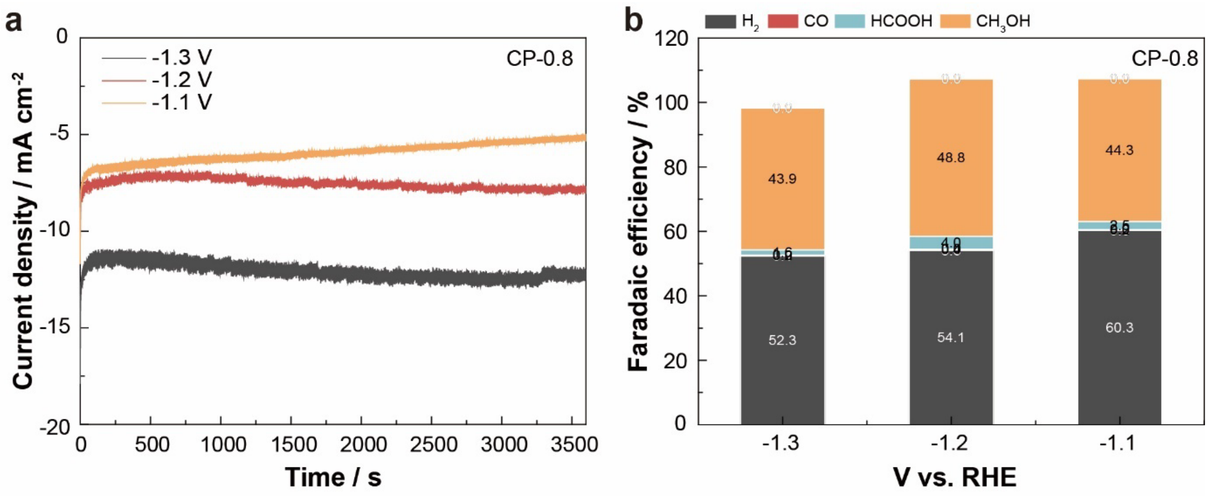

Figure S11. Electrochemical CO2RR using CP-0.8 in 0.1 M $KHCO_3$ electrolyte. (a) Current density profile. (b) Faradaic efficiency. Methanol was the main product across the entire potential range, with a high selectivity of 48.8% measured at -1.2V vs. RHE.

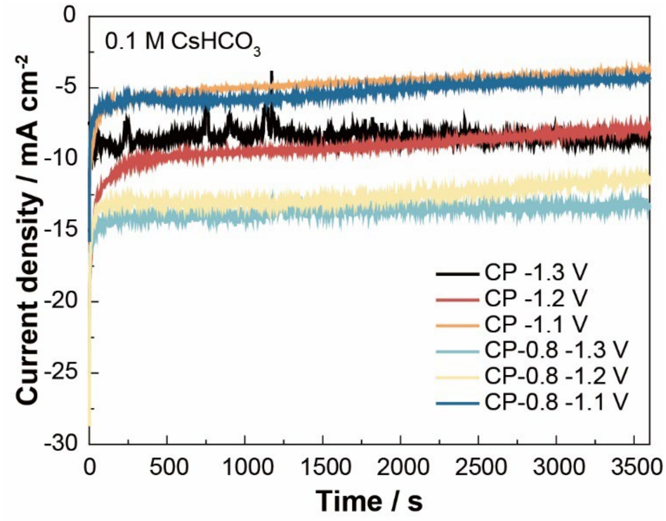

Figure S12. Electrochemical CO2RR using pristine CP and CP-0.8 in 0.1 M $CsHCO_3$ electrolyte at various applied potentials. CP-0.8 exhibited a higher current density than pristine CP.

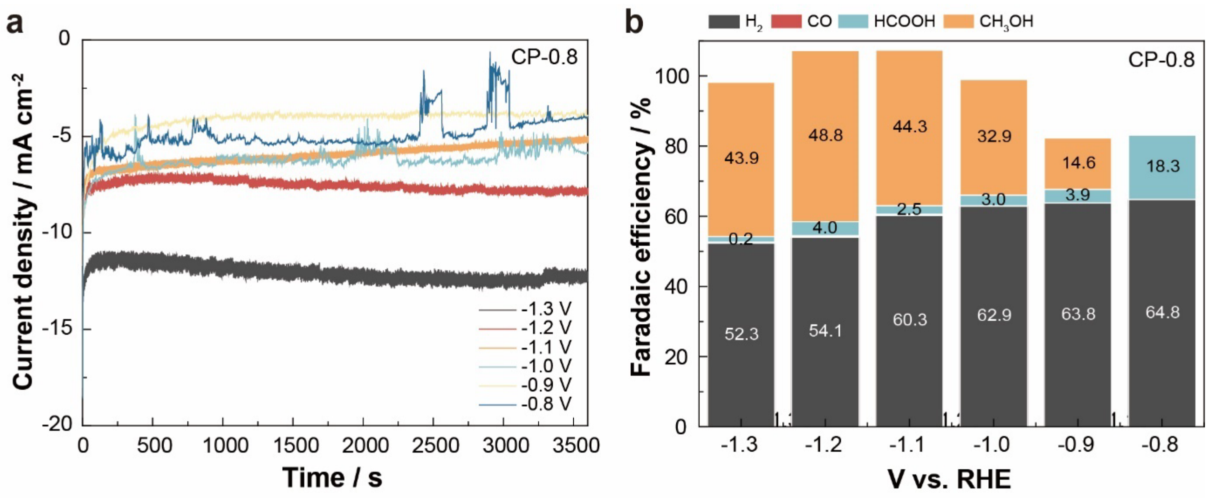

Figure S13. Electrochemical CO2RR using CP-0.8 across a wide range of applied potentials in 0.1 M $KHCO_3$ electrolyte. (a) Current density profile. (b) Faradaic efficiency. Methanol was the main product at higher potentials, while formic acid became the main product at lower potentials.

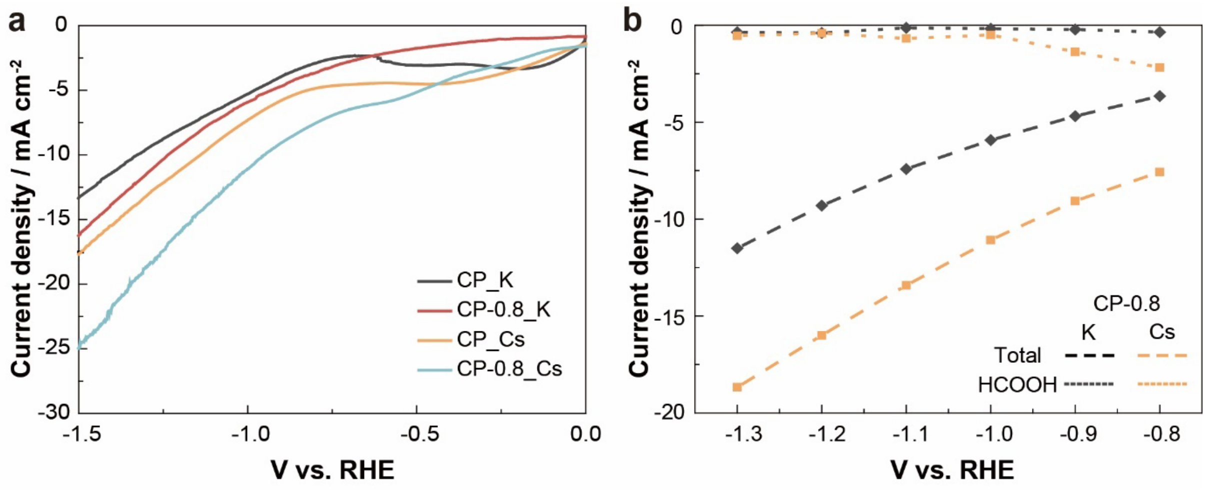

Figure S14. Effect of electrolyte type on CO2RR using pristine CP and CP 0.8. (a) Linear sweep voltammograms (LSV) in 0.1 M $KHCO_3$ (K) or 0.1 M $CsHCO_3$ (Cs) electrolyte. (b) Total current density (dashed) and partial current density for formic acid (dotted).

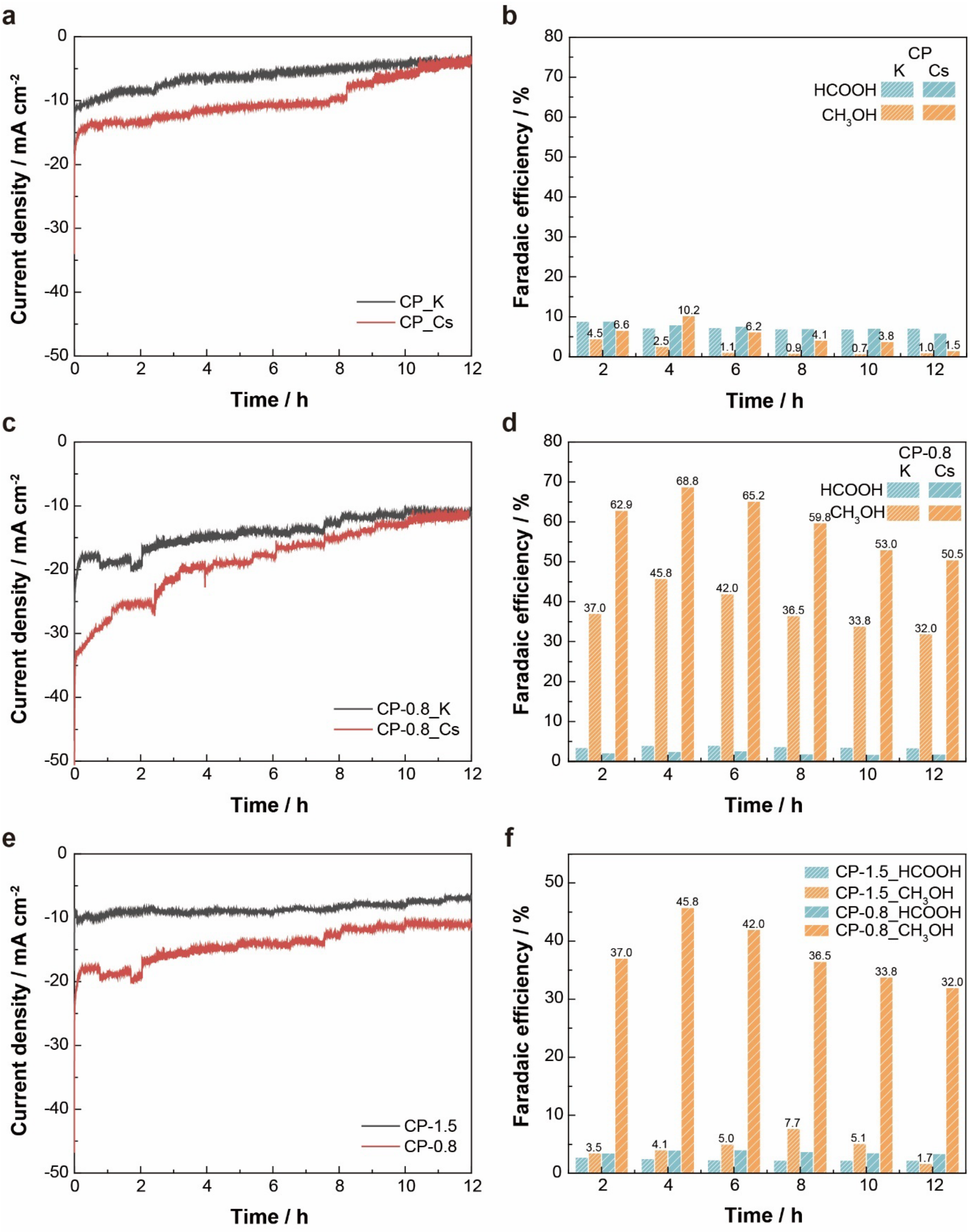

Figure S15. Prolonged CO2RR using pristine CP and CP-0.8 at -1.2 V vs. RHE in 0.1 M $KHCO_3$ (K) or 0.1 M $CsHCO_3$ (Cs). (a-f) Current density profiles (a, c, e) and Faradaic efficiency (b, d, e) for pristine CP (a, b), CP-0.8 (c, d), and CP-1.5 (e, f) for 12 h.

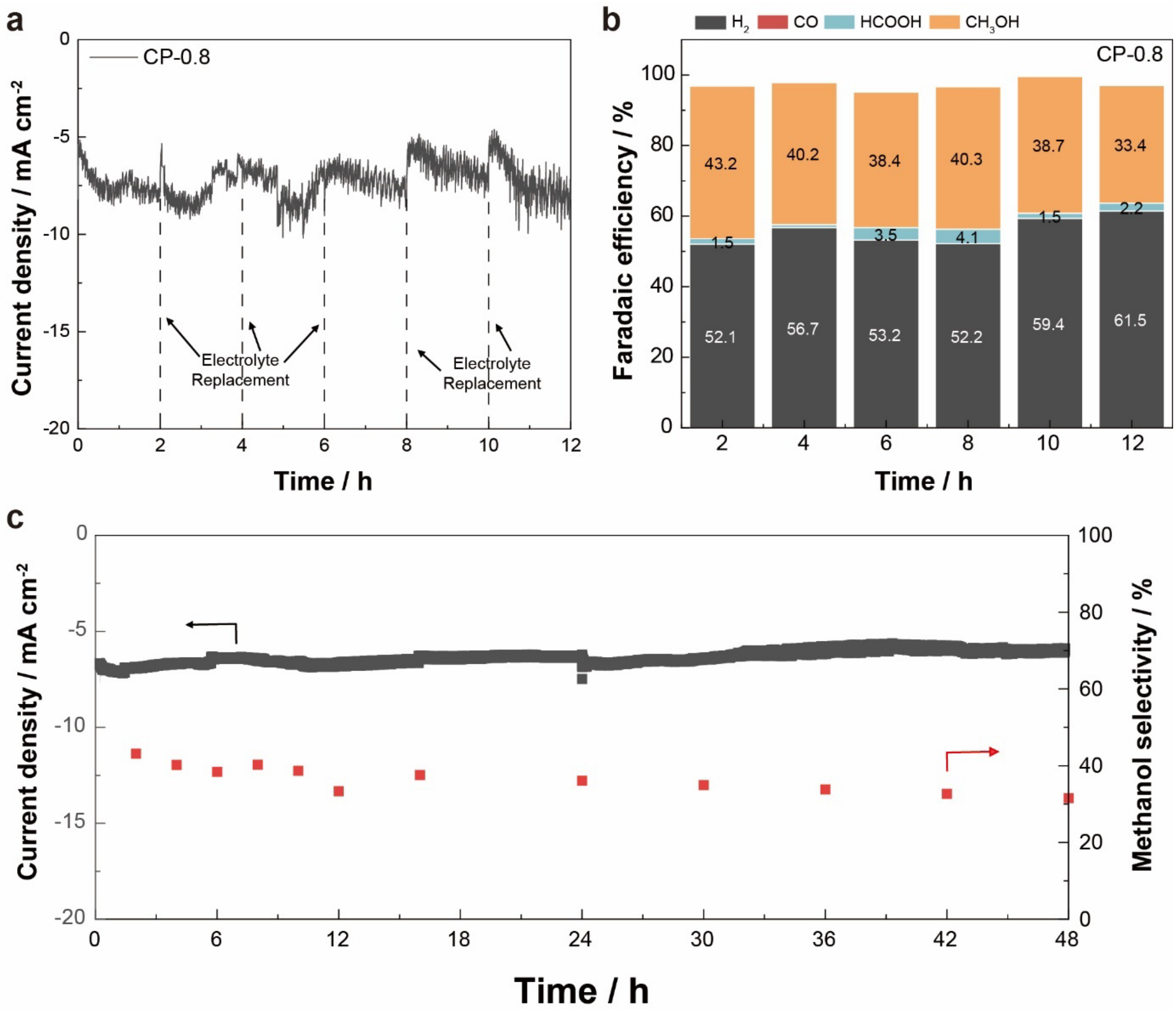

Figure S16. Long-term stability of CP-0.8 for CO2RR to methanol at -1.2 V vs. RHE in 0.1 M $KHCO_3$. (a, b) Current density profile (a) and Faradaic efficiency (b) of CP with electrolyte refreshment and CO_2 purging every 2 h. (c) CO2RR to methanol using CP-0.8 with periodic CO_2 purging every 12 h.

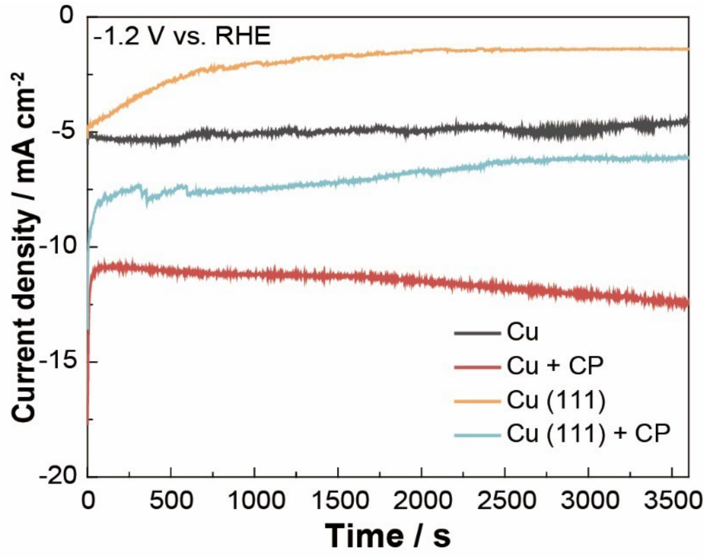

Figure S17. Current density profiles of polycrystalline Cu and single-crystalline Cu (111) with and without pristine CP. This test was conducted at -1.2 V vs. RHE in 0.1 M $KHCO_3$ electrolyte. The coexistence of Cu (111) and CP exhibited a higher current density than the other configurations.

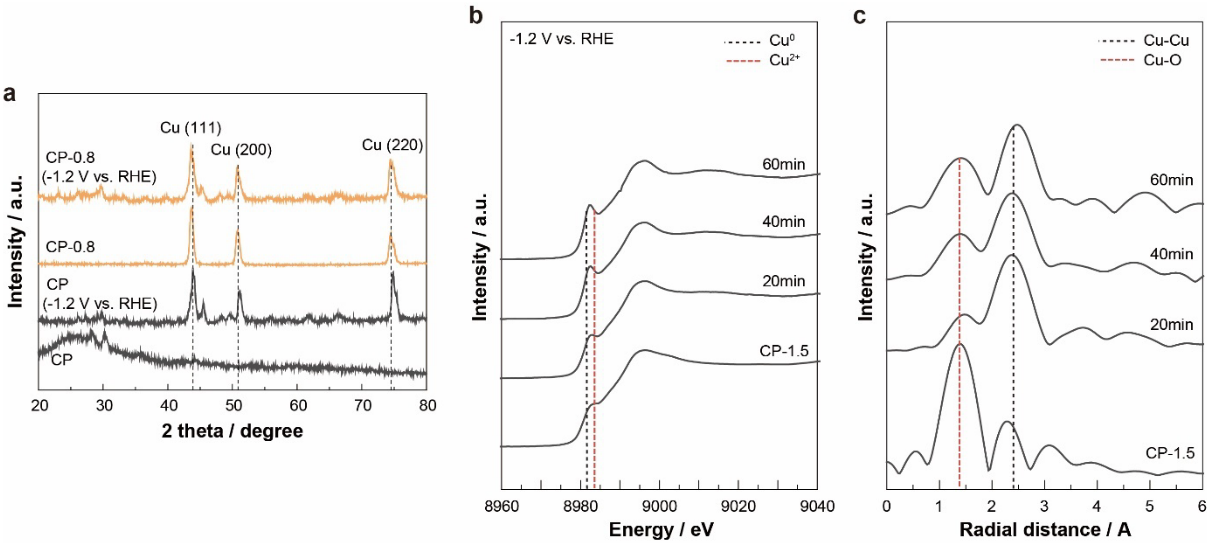

Figure S18. Ex-situ XRD analysis and in-situ X-ray absorption spectroscopy of pristine CP and discharged CP samples. (a) XRD patterns of pristine CP and CP-0.8 before and after CO2RR at -1.2 V vs. RHE in 0.1 M $KHCO_3$. (b, c) In-situ XAFS spectra of CP-1.5 at -1.2 V vs. RHE in 0.1 M $KHCO_3$ electrolyte for 1h at 20 min intervals. Cu-O bonds were rapidly converted to Cu-Cu within 20 min after the electrochemical reaction, indicating low stability for CO2RR.

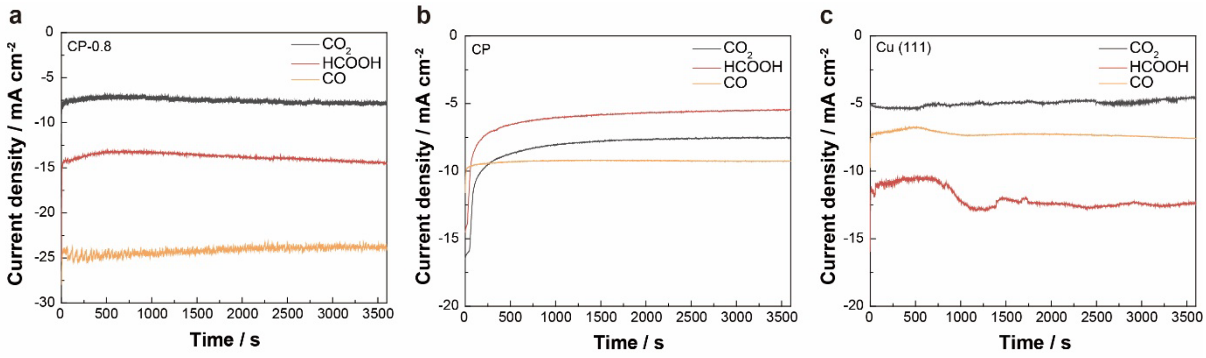

Figure S19. Current density profiles of various catalysts with different feedstocks. (a-c) Current density profiles of CP-0.8 (a), pristine CP (b), and Cu (111) (c) at -1.2 V vs. RHE in 0.1 M $KHCO_3$ electrolyte with different feedstocks (CO_2, HCOOH and CO).

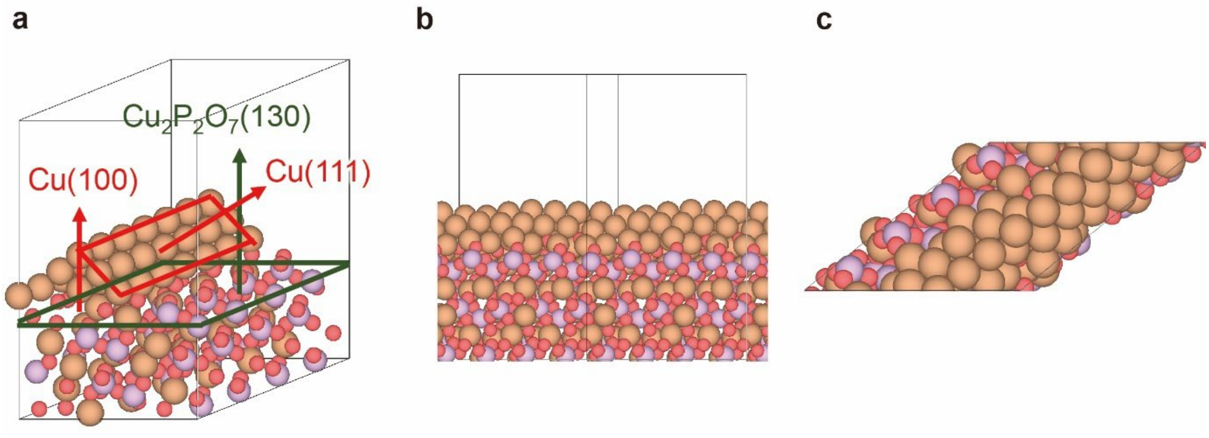

Figure S20. Structural image of the Cu (111) + $Cu_2P_2O_7$ (130) interface. (a) Surface direction before optimizing interface. (b) Side view of the optimized interface. (c) Top view of the optimized interface.

a

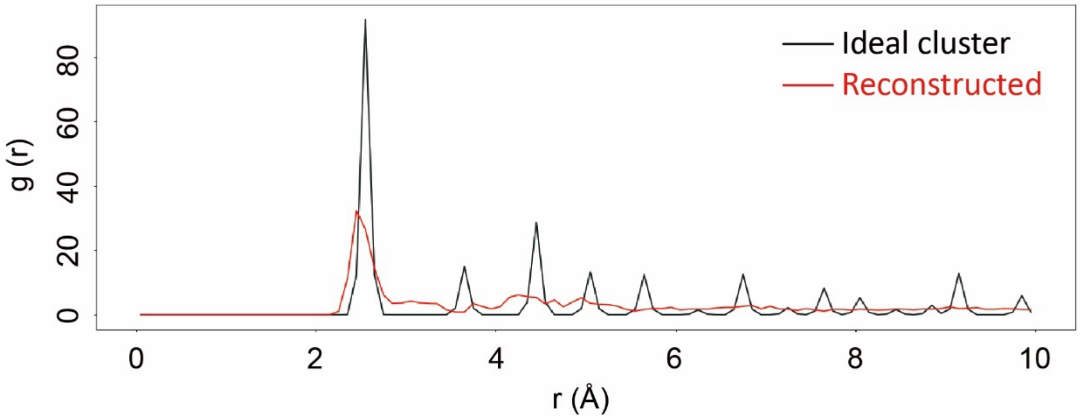

Figure S21. Radial distribution function of Cu-Cu. The black and red lines represent the ideal Cu (111) and the reconstructed Cu (111) slab of Cu (111) + $Cu_2P_2O_7$ (130), respectively.

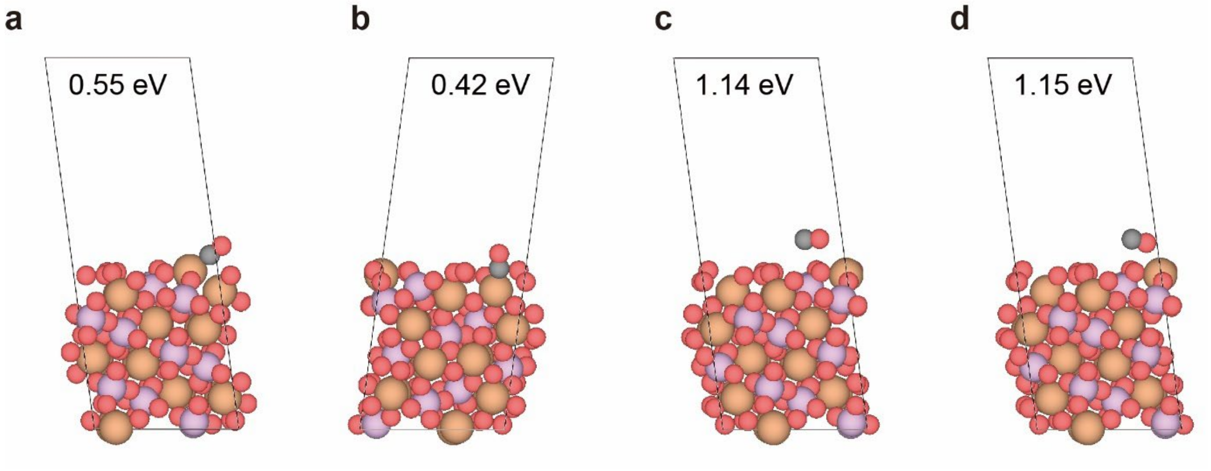

Figure S22. CO adsorption on $Cu_2P_2O_7$ (130). (a-d) Optimized structure with different adsorption sites. The Gibbs energy for each CO adsorption is reported within the figures.

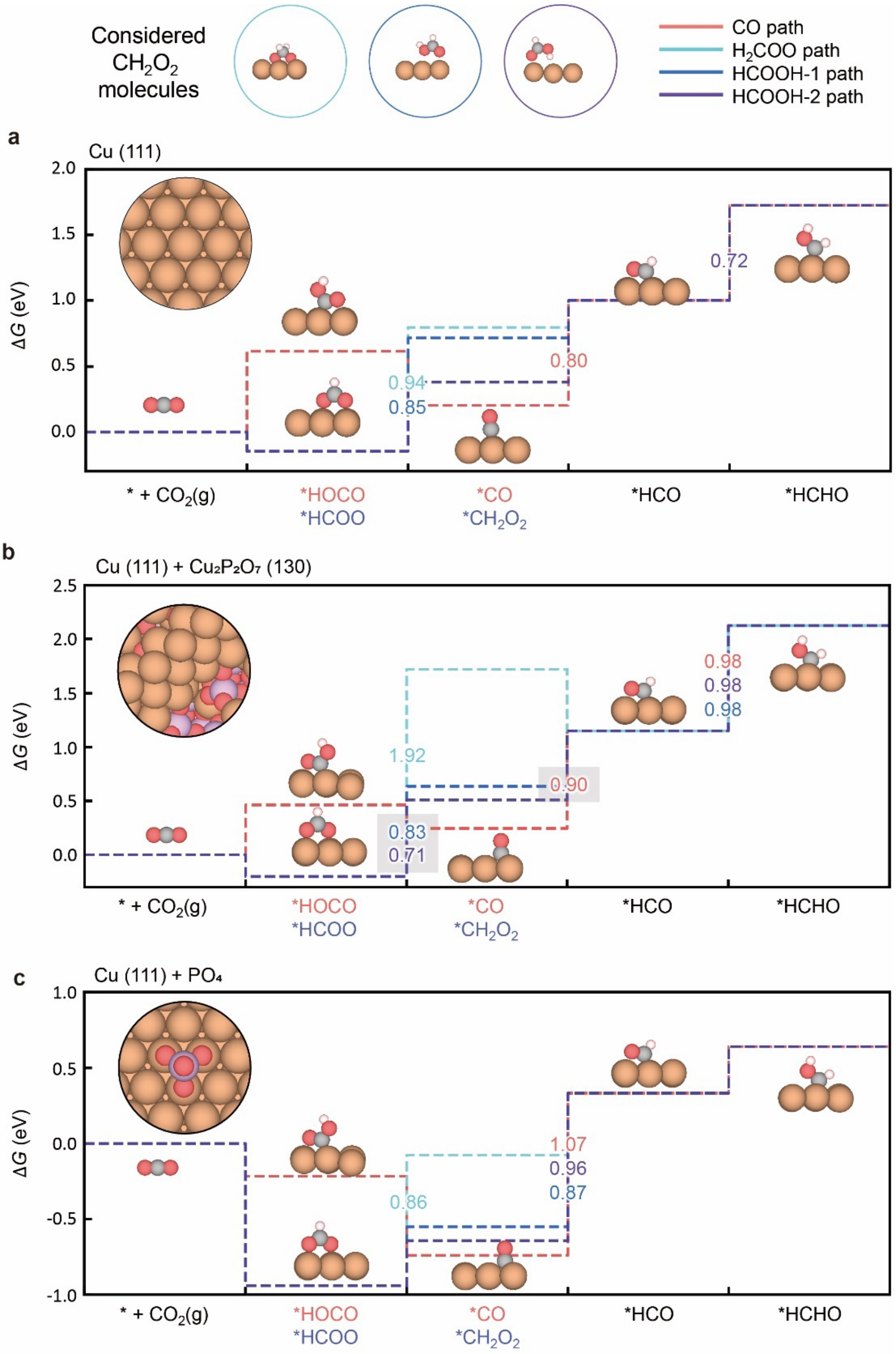

Figure S23. DFT-calculated Gibbs energy diagram for the CO and HCOOH pathways on Cu (111) with and without PO_4. (a, b, c) The Gibbs energy for the CO pathway (red line) and CH_2O_2 pathway (blue-toned line) of Cu (111) (a), Cu (111) + $Cu_2P_2O_7$ (130) (b), and Cu (111) + PO_4 (c). Gibbs energy values for the rate-determining steps are reported within the figure. The second-highest Gibbs energy values are shaded in grey. The Cu, O, C, H, and P atoms are depicted in orange, red, grey, white, and purplish grey, respectively.

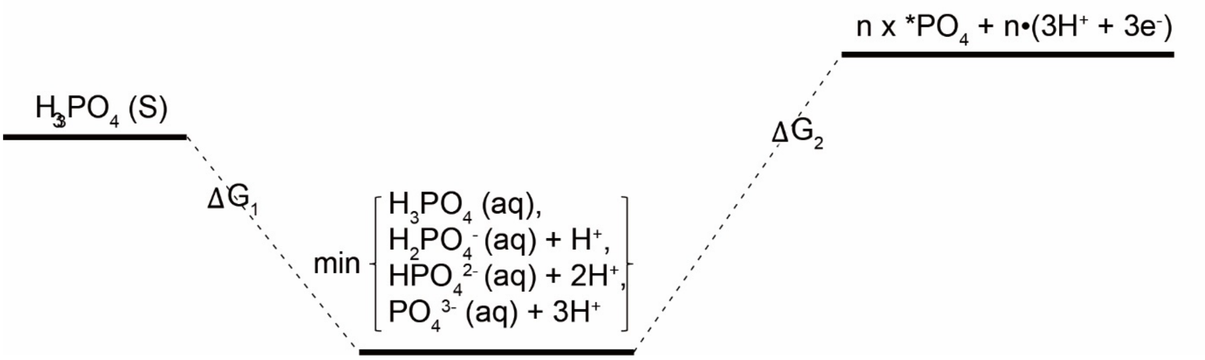

Figure S24. Schematic energy diagram for PO_4 adsorption.

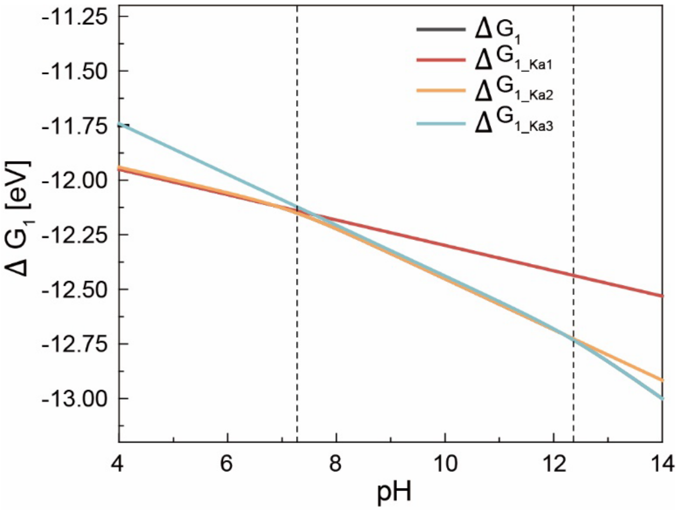

Figure S25. Gibbs energy of phosphate ion formation. The black dotted line represents the minimum Gibbs energy among various configurations. The Gibbs energy values of ΔG_1 and ΔG_{1_Ka1} are identical.

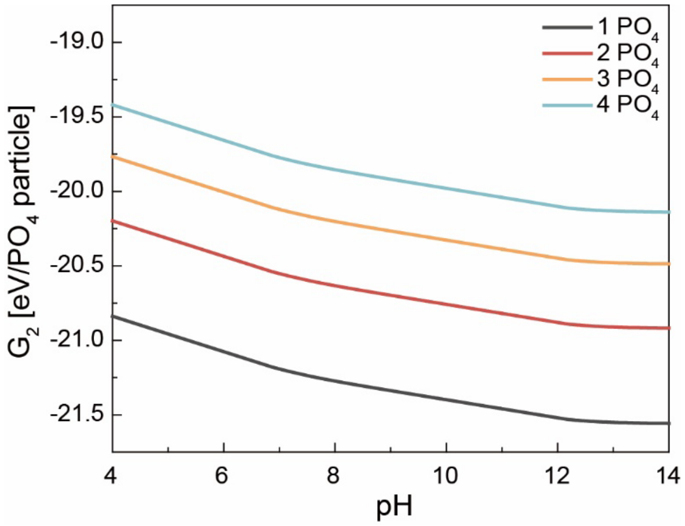

Figure S26. Gibbs energy of 1-4 PO_4 adsorptions on (4 × 4) Cu (111) surface.

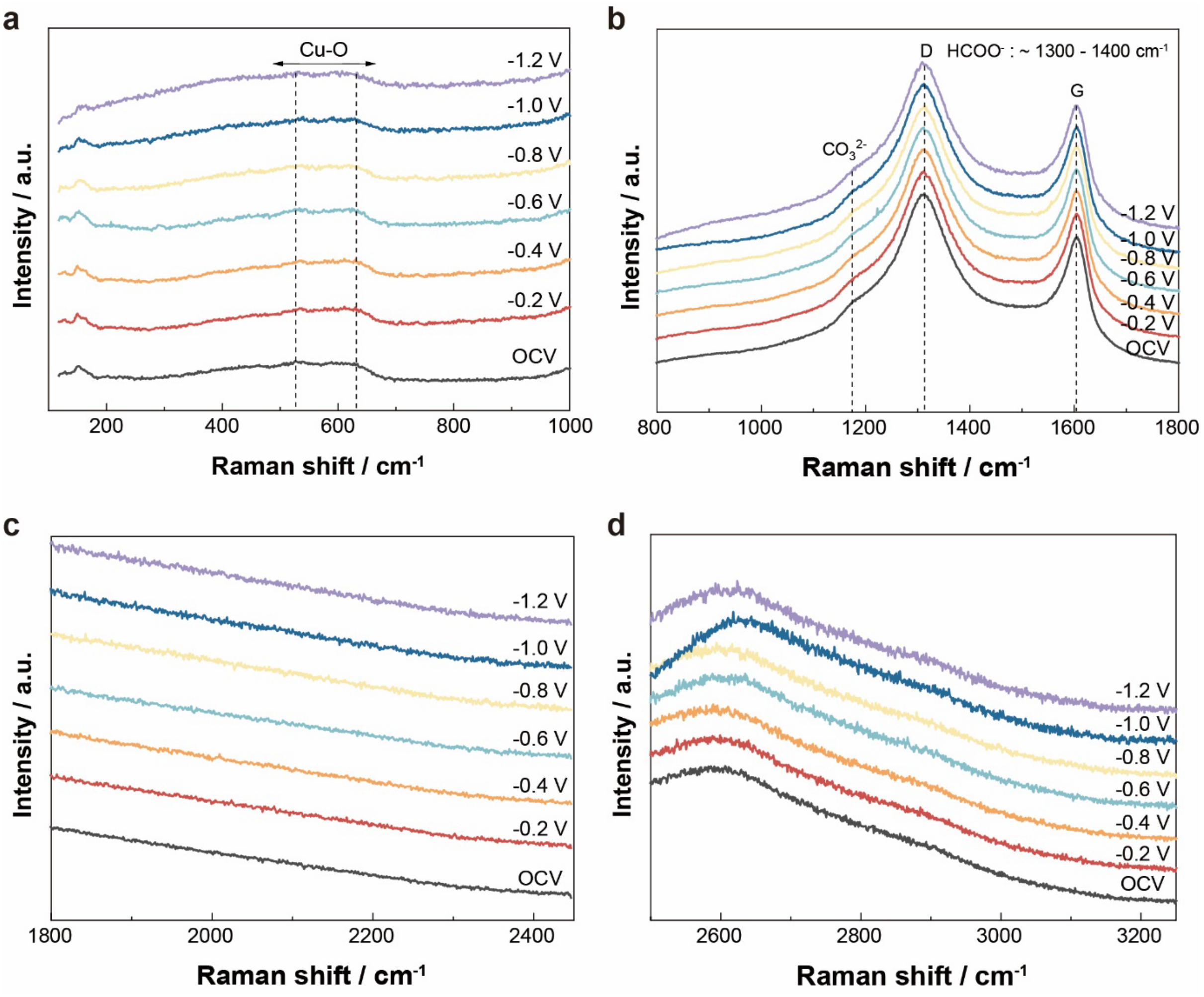

Figure S27. *Operando* Raman spectra of the CP-0.8 catalyst under various applied potentials. (a-d) Raman spectra in different spectral regions: (a) 100–1000 cm^{-1}, showing Cu-O vibration modes; 800–1800 cm^{-1}, indicating peaks corresponding to carbonate (CO_3^{2-}), carbon D-band (~1350 cm^{-1}), and G-band (~1590 cm^{-1}); 1800–400 cm^{-1}; and (d) 2500–3200 cm^{-1}, showing no distinct peaks.

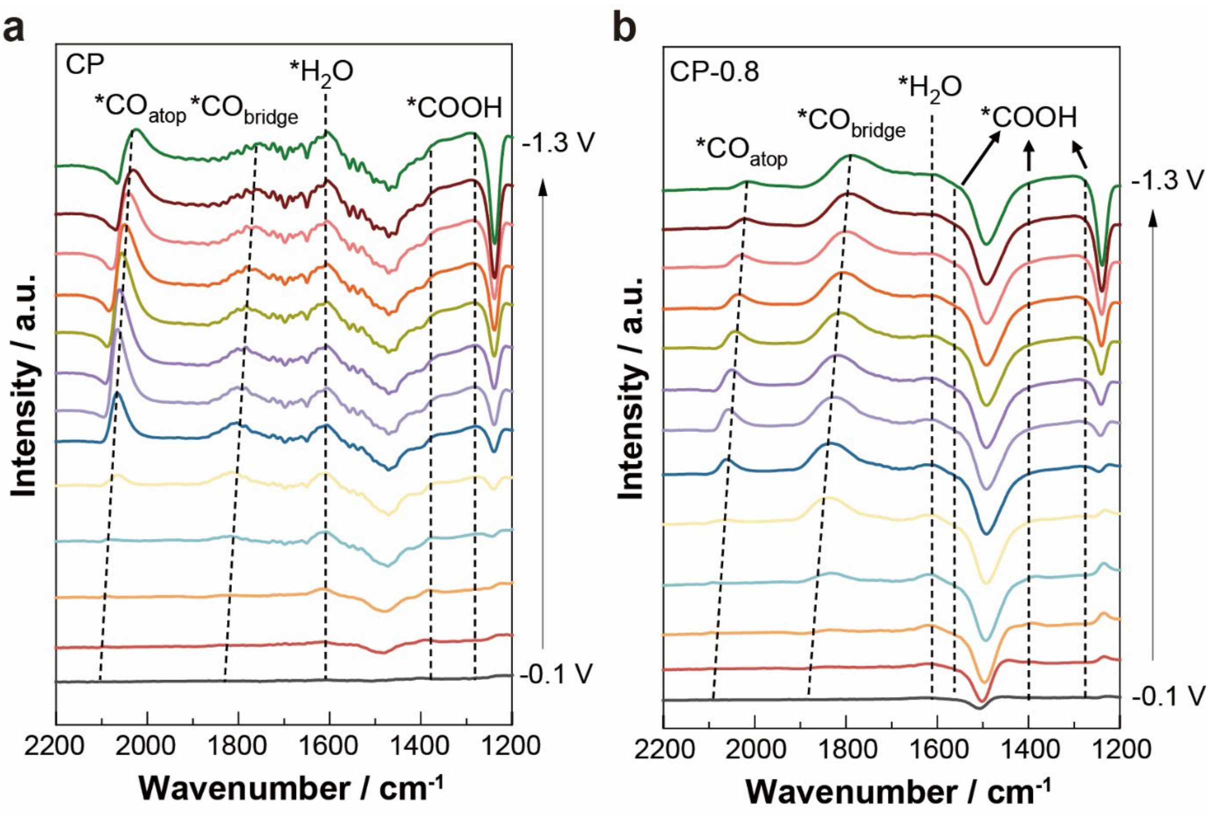

Figure S28. *In situ* IR spectra of CP and CP-0.8 catalysts under various applied potentials. The observed absorption bands correspond to adsorbed intermediates: atop-bound CO (*CO$_{atop}$, 2030–2070 cm^{-1}), bridge-bound CO (*CO$_{bridge}$, 1750–1850 cm^{-1}), adsorbed water (*H_2O, ~ 1640 cm^{-1}), and adsorbed formate species (*COOH, ~1280, 1390, and 1570 cm^{-1}). Dashed lines and arrows indicate these characteristic bands.

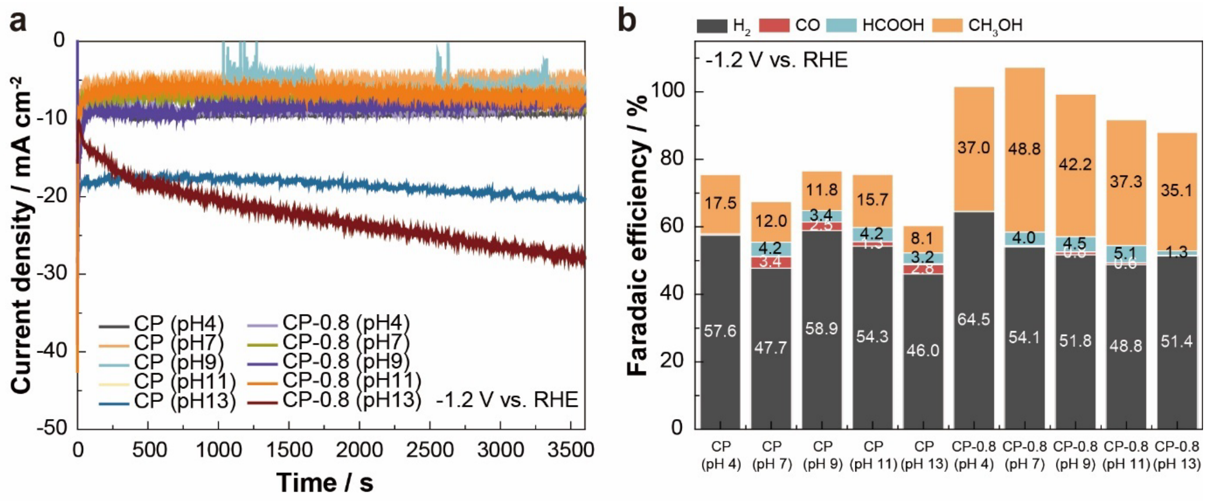

Figure S29. CO2RR activity of pristine CP and CP-0.8 under various pH conditions. (a) Current density profile. (b) Faradaic. Electrolytes used were 0.1 M CH_3COOK for pH 4.0, 0.1 M $KHCO_3$ for pH 7.0, and 0.1 M KOH for pH 13.0.

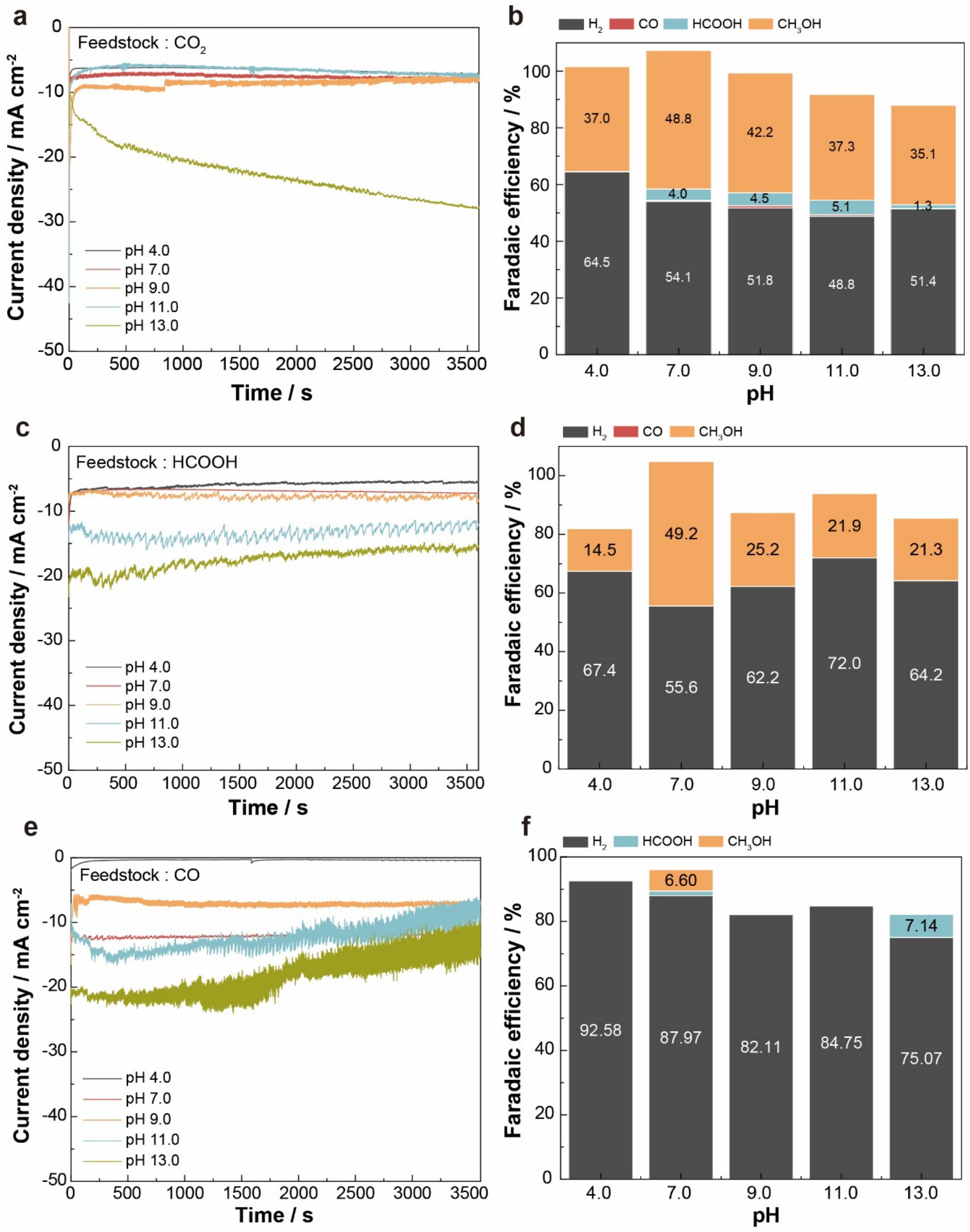

Figure S30. CO2RR using CP-0.8 at -1.2 V vs. RHE in a 0.1 M KHCO3 electrolyte with different feedstocks: (a, b) CO_2, (c, d) HCOOH, and (e, f) CO.

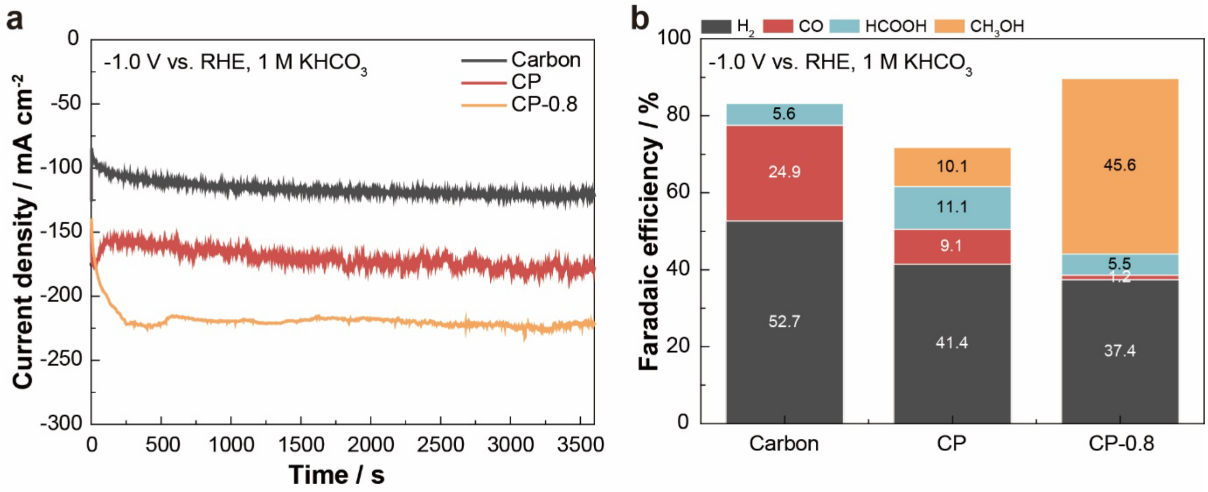

Figure S31. Electrochemical CO2RR using GDEs in flow cells. (a) Current density profile. (b) Faradaic efficiency. Pristine CP and CP-0.8 were coated on a porous carbon-based GDE electrode and tested at -1.0 V vs. RHE in 1 M $KHCO_3$ electrolyte.

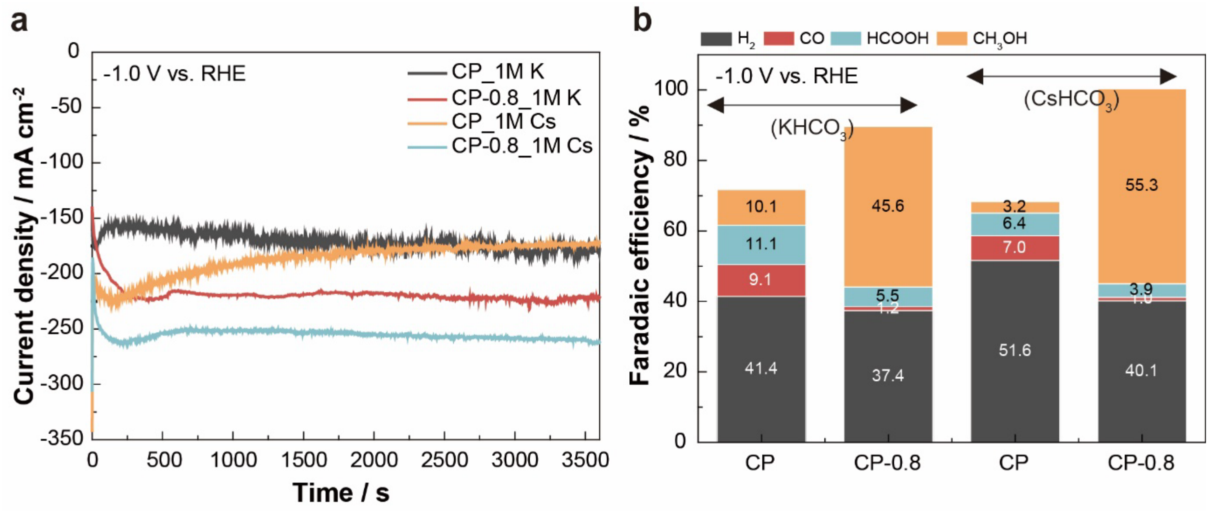

Figure S32. Effect of electrolyte type on electrochemical CO2RR performance with pristine CP and CP-0.8 in GDE cells. (a) Current density profile. (b) Faradaic efficiency. CO2RR test were conducted at -1.0 V vs. RHE in different electrolytes: K = 1 M $KHCO_3$ and Cs = 1 M $CsHCO_3$.

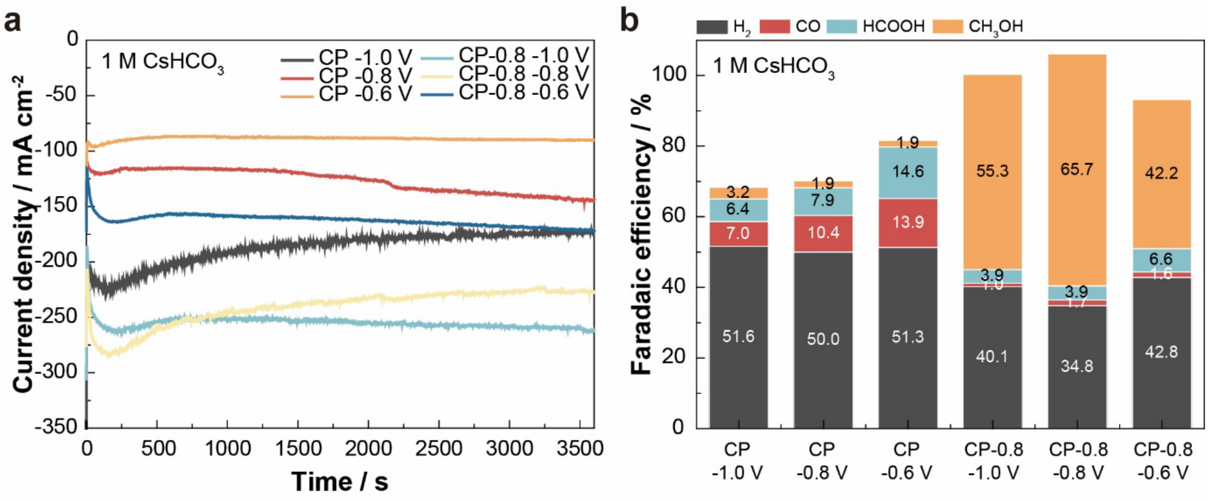

Figure S33. Electrochemical CO2RR using pristine CP and CP-0.8 at various potentials in GDE cells with 1 M $CsHCO_3$. (a) Current density profiles. (b) Faradaic efficiency.

Table S1. Comparison of formic acid production by phosphate or phosphorus-based catalysts via CO2RR.

Catalyst	Electrolyte	Current density of formic acid (Applied potential)	Faradaic efficiency / %	Ref.
OD-PIn	0.5 M $KHCO_3$	-200 mA/cm^2 (-1.2 V vs. RHE)	92.10	[S5]
PO_4^{3-}-In NPs	1 M KOH	-182.80 mA/cm^2	91.40	[S6]
PD-Bi	1 M KOH	-551.40 mA/cm^2	91.90	[S7]
Cu-phosphate complexes	MeCN	-1.45 V vs. NHE	80.00	[S8]

Table S2. Comparison of methanol production by phosphate or phosphorus-based catalysts via CO2RR.

Catalyst	Electrolyte	Current density of methanol (Applied potential)	Faradaic efficiency / %	Ref.
$Cu_3P@C$	1 M KOH	-0.36 V vs. RHE	61.20	[S9]
BP/CP	0.1 M $KHCO_3$	-0.5 V vs. RHE	92.00	[S10]
FeP	0.5 M $KHCO_3$	-0.2 V vs. RHE	80.20	[S11]
$Fe_2P_2S_6$	0.5 M $KHCO_3$	-0.2 V vs. RHE	65.20	[S12]

Table S3. Faradaic efficiency of CO2RR using pristine CP and various discharged CP samples at -1.2 V vs. RHE for 1 h in 0.1 M $KHCO_3$ electrolyte. In all tables below, the chemical formulas CH_3OH, C_2H_5OH, and C_3H_7OH are represented as MeOH, EtOH, and PrOH, respectively.

Sample	Potential V vs. RHE	Products / %								
		H_2	CO	CH_4	C_2H_4	C_2H_6	HCOOH	MeOH	EtOH	PrOH
CP	-1.3	69.50	0.00	0.00	0.00	0.00	3.80	19.20	0.00	0.00
	-1.2	69.80	0.00	0.00	0.00	0.00	1.50	23.40	0.00	0.00
	-1.1	72.20	0.00	0.00	0.00	0.00	3.50	19.30	0.00	0.00
	-1.0	57.88	5.74	0.00	0.00	0.00	4.55	0.00	0.00	0.00
	-0.9	63.10	6.10	0.00	0.00	0.00	4.46	0.00	0.00	0.00
	-0.8	66.14	3.61	0.00	0.00	0.00	3.95	0.00	0.00	0.00
CP-0.8	-1.3	52.34	0.08	0.20	0.00	0.00	1.63	43.94	0.00	0.00
	-1.2	54.08	0.03	0.40	0.00	0.00	3.95	48.83	0.00	0.00
	-1.1	60.29	0.02	0.24	0.00	0.00	2.48	44.31	0.00	0.00
	-1.0	62.91	0.10	0.00	0.00	0.00	3.05	32.91	0.00	0.00
	-0.9	63.79	0.00	0.00	0.00	0.00	3.89	14.65	0.00	0.00
	-0.8	66.09	0.00	0.00	0.00	0.00	7.10	0.00	0.00	0.00
CP-1.5	-1.3	52.06	0.37	0.24	0.00	0.00	2.81	10.35	0.00	0.00
	-1.2	52.95	0.26	0.26	0.00	0.00	2.45	16.14	0.00	0.00
	-1.1	57.45	0.48	0.28	0.00	0.00	3.99	13.53	0.00	0.00
CP-1.2	-1.3	55.36	0.01	0.19	0.00	0.00	2.14	20.59	0.00	0.00
	-1.2	56.93	0.02	0.12	0.00	0.00	1.18	24.46	0.00	0.00
	-1.1	54.78	0.04	0.00	0.00	0.00	3.24	21.79	0.00	0.00
CP-1.0	-1.3	54.57	0.12	0.25	0.00	0.00	3.08	40.58	0.00	0.00
	-1.2	59.33	0.07	0.37	0.00	0.00	4.57	42.51	0.00	0.00
	-1.1	61.52	0.03	0.75	0.00	0.00	1.66	28.13	0.00	0.00

Table S4. Faradaic efficiency of CO2RR using pristine CP and CP-0.8 samples at -1.2 V vs. RHE for 1 h in 0.1 M $CsHCO_3$ electrolyte.

Sample	Potential V vs. RHE	Products / %								
		H_2	CO	CH_4	C_2H_4	C_2H_6	HCOOH	MeOH	EtOH	PrOH
CP	-1.3	50.17	5.09	0.26	0.00	0.00	5.18	5.06	0.00	0.00
	-1.2	49.56	3.22	0.23	0.00	0.00	4.97	12.19	0.00	0.00
	-1.1	51.60	6.10	0.00	0.00	0.00	6.03	13.04	0.00	0.00
CP-0.8	-1.3	41.76	0.27	0.00	0.00	0.00	2.84	51.26	0.00	0.00
	-1.2	32.26	0.07	0.00	0.00	0.00	2.60	66.09	0.00	0.00
	-1.1	32.02	0.18	0.00	0.00	0.00	5.49	70.12	0.00	0.00

Table S5. Faradaic efficiency of long-term CO2RR test for liquid products using pristine CP and discharged CP at -1.2 V vs. RHE in 0.1 M $KHCO_3$ electrolyte.

Sample	Time (Hour)	Products / % HCOOH	MeOH	EtOH	PrOH
CP	2	8.87	4.47	0.00	0.00
	4	7.15	2.54	0.00	0.00
	6	7.27	1.05	0.00	0.00
	8	6.98	0.85	0.00	0.00
	10	6.94	0.67	0.00	0.00
	12	7.11	0.97	0.00	0.00
CP-1.5	2	2.78	3.51	0.00	0.00
	4	2.56	4.05	0.00	0.00
	6	2.34	5.03	0.00	0.00
	8	2.28	7.70	0.00	0.00
	10	2.24	5.14	0.00	0.00
	12	2.24	1.71	0.00	0.00
CP-0.8	2	3.49	37.05	0.00	0.00
	4	4.01	45.76	0.00	0.00
	6	4.06	42.00	0.00	0.00
	8	3.72	36.50	0.00	0.00
	10	3.54	33.82	0.00	0.00
	12	3.41	31.96	0.00	0.00

Table S6. Faradaic efficiency of long-term CO2RR test for liquid products using pristine CP and discharged CP at -1.2 V vs. RHE in 0.1 M $CsHCO_3$ electrolyte.

Sample	Time	Products / %			
	(Hour)	HCOOH	MeOH	EtOH	PrOH
CP	2	8.92	6.61	0.00	0.00
	4	7.98	10.22	0.00	0.00
	6	7.63	6.17	0.00	0.00
	8	7.02	4.10	0.00	0.00
	10	7.09	3.77	0.00	0.00
	12	5.92	1.48	0.00	0.00
CP-0.8	2	2.16	62.87	0.00	0.00
	4	2.54	68.79	0.00	0.00
	6	2.66	65.16	0.00	0.00
	8	1.90	59.77	0.00	0.00
	10	1.78	53.03	0.00	0.00
	12	1.88	50.50	0.00	0.00

Table S7. Faradaic efficiency during the extended stability test of the CP-0.8 catalyst at -1.2 V vs. RHE in 0.1 M $KHCO_3$ electrolyte. H_2 and HCOOH were measured only up to 12h.

Sample	Time	Product / %		
	(Hour)	H_2	HCOOH	CH_3OH
CP-0.8	2	52.10	1.50	43.20
	4	56.70	-	40.20
	6	53.20	3.50	38.40
	8	52.20	4.10	40.30
	10	59.40	1.50	38.70
	12	61.50	2.20	33.40
	16	-	-	37.60
	24	-	-	36.10
	30	-	-	34.98
	36	-	-	33.85
	42	-	-	32.73
	48	-	-	31.60

Table S8. XPS elemental analysis of pristine CP and various discharged CP samples. The relative intensity was calculated in an atomic-weighted manner.

Sample	Elements / %		
	Cu^{2+} or Cu^{1+}	Cu^0	P
Pristine CP	87.67	0.00	12.33
CP-1.5	61.38	31.00	7.62
CP-1.0	43.60	51.88	4.52
CP-0.8	23.12	73.25	3.63

Table S9. Faradaic efficiency of CO2RR using polycrystalline Cu and Cu (111) with and without pristine CP at -1.2 V vs. RHE for 1 h in 0.1 M $KHCO_3$ electrolyte.

Sample	Products / %								
	H_2	CO	CH_4	C_2H_4	C_2H_6	HCOOH	MeOH	EtOH	PrOH
Cu	29.33	5.77	28.43	5.03	0.00	8.79	0.00	0.00	0.00
Cu + CP	26.36	1.70	11.87	19.09	0.00	9.28	9.87	0.00	0.00
Cu (111)	41.99	22.09	25.30	4.76	0.00	16.00	0.00	0.00	0.00
Cu (111) + CP	49.43	2.60	9.65	0.85	0.00	8.22	35.75	0.00	0.00

Table S10. Faradaic efficiency of CO2RR using CP-0.8, pristine CP and Cu (111) at -1.2 V vs RHE for 1 h in 0.1 M $KHCO_3$ electrolyte with different feedstocks (CO_2, HCOOH and CO).

Sample	Feedstock	Products / %								
		H_2	CO	CH_4	C_2H_4	C_2H_6	HCOOH	MeOH	EtOH	PrOH
CP-0.8	CO_2	54.08	0.03	0.40	0.00	0.00	3.95	48.83	0.00	0.00
	HCOOH	55.59	0.28	0.00	0.00	0.00	-	49.19	0.00	0.00
	CO	87.97	-	0.00	0.00	0.00	1.49	6.60	0.00	0.00
CP	CO_2	71.35	2.28	0.06	0.39	0.00	3.91	49.19	0.00	0.00
	HCOOH	70.71	1.31	0.83	1.43	0.00	-	22.63	0.00	0.00
	CO	95.21	-	0.12	00.00	0.00	1.43	3.67	0.00	0.00
Cu (111)	CO_2	45.22	0.25	0.00	0.00	0.00	0.00	0.00	0.00	0.00
	HCOOH	83.41	0.15	3.71	0.19	0.00	-	0.00	0.00	0.00
	CO	76.22	-	2.54	00.00	0.00	1.00	0.00	0.00	0.00

Table S11. Faradaic efficiency of CO2RR using pristine CP and CP-0.8 at -1.2 V vs. RHE for 1 h under different pH conditions. 0.1 M CH_3COOK, 0.1 M $KHCO_3$, and 0.1 M KOH electrolytes were used for pH 4.0, 7.0-11.0, and 13.0, respectively.

Sample	pH	Products / %								
		H_2	CO	CH_4	C_2H_4	C_2H_6	HCOOH	MeOH	EtOH	PrOH
CP	4.0	57.55	0.41	0.00	0.00	0.00	0.00	17.51	0.00	0.00
	7.0	47.75	3.42	0.00	0.00	0.00	4.21	12.02	0.00	0.00
	9.0	58.94	2.53	0.00	0.00	0.00	3.36	11.78	0.00	0.00
	11.0	54.31	1.30	0.00	0.00	0.00	4.22	15.72	0.00	0.00
	13.0	46.05	2.75	0.00	0.00	0.00	3.21	8.05	0.00	0.00
CP-0.8	4.0	64.49	0.08	0.00	0.00	0.00	0.00	37.02	0.00	0.00
	7.0	54.08	0.03	0.00	0.00	0.00	3.95	48.83	0.00	0.00
	9.0	51.76	0.81	0.00	0.00	0.00	4.55	42.21	0.00	0.00
	11.0	48.79	0.59	0.00	0.00	0.00	5.09	37.28	0.00	0.00
	13.0	51.40	0.05	0.00	0.00	0.00	1.35	35.14	0.00	0.00

Table S12. Faradaic efficiency of CO2RR using pristine CP and CP-0.8 on a GDE electrode at -1.0 V vs. RHE for 1 h in 1 M $KHCO_3$ electrolyte.

Sample	Products / %								
	H_2	CO	CH_4	C_2H_4	C_2H_6	HCOOH	MeOH	EtOH	PrOH
Carbon (C)	52.66	24.90	0.00	0.00	0.00	5.64	0.00	0.00	0.00
CP	41.43	9.07	0.00	0.00	0.00	11.11	10.13	0.00	0.00
CP-0.8	37.40	1.17	0.00	0.00	0.00	5.49	45.59	0.00	0.00

Table S13. Faradaic efficiency of CO2RR using pristine CP and CP-0.8 on a GDE electrode at -1.0 V vs. RHE for 1 h in 1 M $KHCO_3$ or 1 M $CsHCO_3$ electrolyte.

Sample (Electrolyte)	Products / %								
	H_2	CO	CH_4	C_2H_4	C_2H_6	HCOOH	MeOH	EtOH	PrOH
CP ($KHCO_3$)	41.43	9.07	0.00	0.00	0.00	11.11	10.13	0.00	0.00
CP-0.8 ($KHCO_3$)	37.40	1.17	0.00	0.00	0.00	5.49	45.59	0.00	0.00
CP ($CsHCO_3$)	51.62	7.03	0.00	0.00	0.00	6.42	3.23	0.00	0.00
CP-0.8 ($CsHCO_3$)	40.15	1.01	0.00	0.00	0.00	3.86	55.31	0.00	0.00

Table S14. Faradaic efficiency of CO2RR using pristine CP and CP-0.8 on a GDE electrode at various potentials for 1 h in 1 M $CsHCO_3$ electrolyte.

Sample	Potential V vs RHE	Products / %								
		H_2	CO	CH_4	C_2H_4	C_2H_6	HCOOH	MeOH	EtOH	PrOH
CP	-1.0	51.62	7.03	0.00	0.00	0.00	6.42	3.23	0.00	0.00
	-0.8	50.01	10.37	0.00	0.00	0.00	7.85	1.90	0.00	0.00
	-0.6	51.29	13.87	0.00	0.00	0.00	14.65	1.86	0.00	0.00
CP-0.8	-1.0	40.15	1.00	0.00	0.00	0.00	3.86	55.31	0.00	0.00
	-0.8	34.80	1.66	0.00	0.00	0.00	3.95	65.71	0.00	0.00
	-0.6	42.80	1.58	0.00	0.00	0.00	6.62	42.19	0.00	0.00

Table S15. Comparison of electrocatalysts for methanol production via CO2RR in H-cells.

Catalyst	Electrolyte	Current density of methanol (Applied potential)	Faradaic efficiency / %	Stability / h	Ref.
CP-0.8	0.1 M $KHCO_3$	-3.66 mA/cm^2 (-1.2 V vs. RHE)	48.80	48.0	This work
	0.1 M $CsHCO_3$	-9.46 mA/cm^2 (-1.1 V vs. RHE)	70.10	12.0	
Cu/Au/NCF	0.5 M $KHCO_3$	None (-1.0 V vs. RHE)	15.90	None	[S13]
CuSAs/TCNFs	0.1 M $KHCO_3$	-40.92 mA/cm^2 (-0.9 V vs. RHE)	44.00	50.0	[S14]
$Ti_3(Al_{1-x}Cu_x)C_2$	0.1 M $KHCO_3$	-14.76 mA/cm^2 (-1.4 V vs. RHE)	59.10	30.0	[S15]
Cu_2O/ZnO	0.5 M $KHCO_3$	-1.88 mA/cm^2 (-1.3 V vs. Ag/AgCl)	17.70	1.5	[S16]
$CuBi_{12}$	0.5 M $KHCO_3$	-0.86 mA/cm^2 (-0.21 V vs. RHE)	8.60	5.0	[S17]
Cu_3P@C	0.3 M $KHCO_3$	-1.18 mA/cm^2 (-0.36 V vs. RHE)	59.20	24.0	[S10]
$CuGa_2$	0.5 M $KHCO_3$	-0.77 mA/cm^2 (-0.3 V vs. RHE)	77.26	2.0	[S18]
CuO NWs	0.1 M $KHCO_3$	-7.97 mA/cm^2 (-1.4 V vs. Ag/AgCl)	66.40	2.0	[S19]
Cu_2O/CuO-300	0.5 M $KHCO_3$	-2.97 mA/cm^2 (-1.3 V vs. Ag/AgCl)	6.46	2.0	[S20]
$Pd_{83}Cu_{17}$	$BMImBF_4/H_2O$	-25.44 mA/cm^2 (-2.1 V vs. Ag/Ag$^+$)	80.00	24.0	[S21]
ZnO-Cu-C60	$BMImBF_4/H_2O$	-15.89 mA/cm^2 (-0.63 V vs. RHE)	78.30	20.0	[S22]
$Cu_{1.63}Se$	0.1 M MeCN/H_2O	-31.62 mA/cm^2 (-2.1 V vs. Ag/Ag$^+$)	77.60	25.0	[S23]
Ag, S-Cu_2O/Cu	$BMImBF_4/H_2O$ (1:3)	-82.70 mA/cm^2 (-1.2 V vs. RHE)	67.40	24.0	[S24]

Table S16. Comparison of electrocatalysts for methanol production via CO2RR in GDE cells.

Catalyst	Electrolyte	Current density of methanol (Applied potential or Applied current density)	Faradaic efficiency / %	Ref.
CP-0.8	1M $KHCO_3$	-113.30 mA/cm^2 (-1.0 V vs. RHE)	45.60	This work
	1M $CsHCO_3$	-145.52 mA/cm^2 (-0.8 V vs. RHE)	65.70	
CoPC-NH_2/CNT [25]	0.3M $KHCO_3$	-129.40 mA/cm^2 (-300 mA/cm^2)	43.10	[S25]
Cu_3P@C [10]	1M KOH	-130.00 mA/cm^2 (-0.76 V vs. RHE)	40.00	[S10]
$CuGa_2$ [18]	1M KOH	-16.70 mA/cm^2 (-0.3 V vs. RHE)	78.00	[S18]
CuO NWs [19]	1M KOH	-29.75 mA/cm^2 (-45 mA/cm^2)	66.10	[S19]

References

[S1] a) G. Kresse, D. Joubert, *Phys. Rev. B* **1999**, *59*, 1758; b) G. Kresse, J. Furthmüller, *Phys. Rev. B* **1996**, *54*, 11169.

[S2] J. Klimes, D. R. Bowler, A. Michaelides, *J. Phys. Condens. Matter* **2010**, *22*, 022201.

[S3] a) I. C. Man, H. Y. Su, F. Calle-Vallejo, H. A. Hansen, J. I. Martínez, N. G. Inoglu, J. Kitchin, T. F. Jaramillo, J. K. Nørskov, J. Rossmeisl, *ChemCatChem* **2011**, *3*, 1159; b) Y.-J. Tak, S. Yang, H. Lee, D.-H. Lim, A. Soon, *J. Ind. Eng. Chem.* **2018**, *58*, 208.

[S4] P. Sebastián-Pascual, A. S. Petersen, A. Bagger, J. Rossmeisl, M. Escudero-Escribano, *ACS Catal.* **2021**, *11*, 1128.

[S5] F. Jia, X. Yu, L. Zhang, *J. Power Sources* **2014**, *252*, 85.

[S6] Z. Wei, J. Ding, Z. Wang, A. Wang, L. Zhang, Y. Liu, Y. Guo, X. Yang, Y. Zhai, B. Liu, *Angew. Chem. Int. Ed.* **2024**, *63*, e202402070.

[S7] B. Zhang, Y. Chang, P. Zhai, C. Wang, J. Gao, L. Sun, J. Hou, *Adv. Mater.* **2023**, *35*, e2304379.

[S8] Y. Xing, H. Chen, Y. Liu, Y. Sheng, J. Zeng, Z. Geng, J. Bao, *Chem. Commun.* **2021**, *57*, 1502.

[S9] J. Zhao, L. Sun, S. Canepa, H. Sun, M. N. Yesibolati, M. Sherburne, R. Xu, T. Sritharan, J. S. C. Loo, J. W. Ager Iii, J. Barber, K. Mølhave, Z. J. Xu, *J. Mater. Chem. A* **2017**, *5*, 11905.

[S10] H. Yu, X. Han, Z. Hua, W. Yang, X. Wu, Y. Wu, S. Chen, W. Hong, S. Deng, J. Zhang, J. Wang, *ACS Catal.* **2024**, *14*, 12783.

[S11] S. Mou, T. Wu, J. Xie, Y. Zhang, L. Ji, H. Huang, T. Wang, Y. Luo, X. Xiong, B. Tang, X. Sun, *Adv. Mater.* **2019**, *31*, e1903499.

[S12] L. Ji, L. Li, X. Ji, Y. Zhang, S. Mou, T. Wu, Q. Liu, B. Li, X. Zhu, Y. Luo, X. Shi, A. M. Asiri, X. Sun, *Angew. Chem. Int. Ed.* **2020**, *59*, 758.

[S13] L. Ji, L. Chang, Y. Zhang, S. Mou, T. Wang, Y. Luo, Z. Wang, X. Sun, *ACS Catal.* **2019**, *9*, 9721.

[S14] H. Yang, Y. Wu, G. Li, Q. Lin, Q. Hu, Q. Zhang, J. Liu, C. He, *J. Am. Chem. Soc.* **2019**, *141*, 12717.

[S15] Q. Zhao, C. Zhang, R. Hu, Z. Du, J. Gu, Y. Cui, X. Chen, W. Xu, Z. Cheng, S. Li, B. Li, Y. Liu, W. Chen, C. Liu, J. Shang, L. Song, S. Yang, *ACS Nano* **2021**, *15*, 4927.

[S16] J. Albo, A. Sáez, J. Solla-Gullón, V. Montiel, A. Irabien, *Appl. Catal. B: Environ* **2015**, *176*, 709.

[S17] J. Albo, M. Perfecto-Irigaray, G. Beobide, A. Irabien, *J. CO_2. Util* **2019**, *33*, 157.

[S18] D. Bagchi, J. Raj, A. K. Singh, A. Cherevotan, S. Roy, K. S. Manoj, C. P. Vinod, S. C. Peter, *Adv. Mater.* **2022**, *34*, e2109426.

[S19] C. Azenha, C. Mateos-Pedrero, M. Alvarez-Guerra, A. Irabien, A. Mendes, *Electrochimica Acta* **2020**, 363, 137207.

[S20] A. Roy, H. S. Jadhav, J. Gil Seo, *Electroanalysis* **2020**, *33*, 705.

[S21] L. Lu, X. Sun, J. Ma, D. Yang, H. Wu, B. Zhang, J. Zhang, B. Han, *Angew. Chem. Int. Ed.* **2018**, *57*, 14149.

[S22] Y. Li, H. Zhang, T. Chen, Y. Sun, F. Rosei, M. Yu, *Adv. Funct. Mater.* **2023**, *34*, 2312970.

[S23] D. Yang, Q. Zhu, C. Chen, H. Liu, Z. Liu, Z. Zhao, X. Zhang, S. Liu, B. Han, *Nat. Commun.* **2019**, *10*, 677.

[S24] P. Li, J. Bi, J. Liu, Q. Zhu, C. Chen, X. Sun, J. Zhang, B. Han, *Nat. Commun.* **2022**, *13*, 1965.

[S25] S. Cheon, J. Li, H. Wang, *J. Am. Chem. Soc.* **2024**, *146*, 16348.
